# Interleukin-1β Enhances FasL-Induced Caspase-3/-7 Activity without Increasing Apoptosis in Primary Mouse Hepatocytes

**DOI:** 10.1371/journal.pone.0115603

**Published:** 2014-12-31

**Authors:** Anna Lutz, Julia Sanwald, Maria Thomas, Ronny Feuer, Oliver Sawodny, Michael Ederer, Christoph Borner, Matjaz Humar, Irmgard Merfort

**Affiliations:** 1 Department of Pharmaceutical Biology and Biotechnology, Albert Ludwigs University Freiburg, Freiburg, Germany; 2 Institute for System Dynamics, University of Stuttgart, Stuttgart, Germany; 3 Dr. Margarete Fischer-Bosch Institute of Clinical Pharmacology, Stuttgart, Germany; 4 Institute of Molecular Medicine and Cell Research, Albert Ludwigs University Freiburg, Freiburg, Germany; 5 Spemann Graduate School of Biology and Medicine (SGBM), Albert Ludwigs University Freiburg, Freiburg, Germany; 6 Bioss – Centre for Biological Signaling Studies, Albert Ludwigs University Freiburg, Freiburg, Germany; Innsbruck Medical University, Austria

## Abstract

Sustained inflammation may increase the susceptibility of hepatocytes to apoptotic cell death and therefore exacerbate liver damage. Here we report that the pro-inflammatory cytokine IL-1β sensitizes primary murine hepatocytes to Fas ligand (FasL)-induced caspase-3/-7 activity. This process was dependent on JNK1/2 and the BH3-only proteins Bim and Bid. Mathematical modeling revealed that incubation of hepatocytes with IL-1β depleted the anti-apoptotic Bcl-2 protein pool and thus shifted hepatocytes to mitochondrial type II apoptosis following Fas activation. As a consequence, IL-1β and FasL treatment enhanced cytochrome c release. Surprisingly, despite increased caspase-3/-7 activation, FasL-induced cell death was reduced by IL-1β pre-treatment. This protective effect was independent of JNK1/2, Bim or Bid. Furthermore, elevated caspase-3/-7 activity upon IL-1β and FasL treatment did not result in enhanced PARP cleavage. The protective effect of IL-1β was seen after 3 h of pre-incubation, indicating an anti-apoptotic transcriptional response. Indeed, NF-κB DNA binding was increased in response to IL-1β plus FasL and gene-expression profiling of NF-κB regulated genes revealed a transcriptional and translational upregulation of the caspase-8 inhibitor A20. A mathematical model was developed to explain the contradictious occurrence of both increased caspase-3/-7 activity and elevated cell viability by including a heterogeneous distribution of Bcl-2 proteins and variations in Fas signaling resulting in different subpopulations of hepatocytes.

## Introduction

Hepatocyte apoptosis is critically involved in the pathogenesis of liver diseases and inflammation and is regarded as one of the driving forces of cirrhosis, the final stage of chronic liver disease. An in-depth understanding of the mechanistic interplay of different soluble and cell-associated apoptotic and pro-inflammatory signals is therefore urgently needed. The maintenance of liver homeostasis is procured by different cytokines such as tumor necrosis factor α (TNFα), interleukin-6 (IL-6), and interleukin-1β (IL-1β), which mediate signaling between the different cell types of the liver organ [1].

In a previous study we showed that pre-incubation with TNFα sensitizes primary murine hepatocytes to Fas ligand (FasL) induced apoptosis [2]. However, the effect of IL-1β in this cellular response is incompletely understood. It was reported that IL-1β protects mice from FasL-induced apoptosis [3], but negatively influenced liver disease [4], [5]. In the liver, IL-1β is released by Kupffer cells. Binding to its cell surface interleukin-1 receptor (IL-1R) [6] results in the activation of different signaling pathways, including those governed by c-Jun, p38-MAPK or NF-κB [7], [8].

FasL binds to its cell surface receptor Fas/CD95 and is a strong inducer of apoptosis. Receptor binding leads to the formation of the death inducing signaling complex (DISC) [9]. In this complex, pro-caspase-8 is cleaved to caspase-8 which can either directly cleave pro-caspase-3 (type I pathway) [10], or cleave the BH3-only protein Bid into its truncated form tBid, activating Bax/Bak-mediated mitochondrial membrane permeabilization (MOMP) (type II pathway). MOMP ensures the release of pro-apoptotic factors like cytochrome c and Smac/DIABLO into the cytosol [11]. Cytochrome c activates the apoptosome comprising Apaf-1 and caspase-9, which finally cleaves and activates the executioner caspase-3 to induce cell death. Smac/DIABLO binds to and impedes the function of the anti-apoptotic X-linked Inhibitor of Apoptosis Protein XIAP, which is an inhibitor of caspase-3 and -9 [12].

TNFα is a pleiotropic cytokine, which usually cannot directly induce apoptosis of mammalian cells, including primary murine hepatocytes, because it simultaneously leads to activation of the survival factor NF-κB [13]. However, our recent findings demonstrated that pre-treating primary hepatocytes cultured on collagen with TNFα sensitizes them to FasL-induced apoptosis, which is mediated via JNK activation and the BH3-only proteins Bim and Bid [2]. The mechanism of TNFα-induced sensitization was supported by a mathematical model [2] that was later extended in Schlatter et al. [14].

In this study, we show that IL-1β also sensitizes primary murine hepatocytes to FasL-induced caspase-3 activation but in contrast to TNFα, this leads to elevated cell viability. As with TNFα, increased caspase-3 activity in response to IL-1β is mediated via JNK activation, Bim and Bid as well as cytochrome c release. Based on the TNFα/FasL model [14] a mathematical model has been developed which is able to reproduce all observed effects of the IL-1β sensitization of caspase-3 activity and, furthermore, provides an explanation for the simultaneous occurrence of both increased caspase-3/-7 activity and cell viability.

## Materials and Methods

### Mice strains and hepatocyte isolation

Wild type (C57BL/6N) and IL-1R^-/-^ mice were purchased from Jackson Laboratories. Bid^-/-^, Bim^-/-^ and XIAP^-/-^ mice were kindly provided by A. Strasser and J. Silke, WEHI, Melbourne. Primary hepatocytes were isolated from 8–14 week old BL6 mice using the collagenase perfusion technique and cultivated as previously described [15]. The whole study with the mice including the isolation procedure was approved by the animal experimental committee (ethical permission number: X-12/22D, University of Freiburg), and animals were handled and housed according to specific pathogen free (SPF) conditions.

### Antibodies, reagents and cell culture consumables

NF-κB oligonucleotide: Promega, Mannheim, Germany; [γ33P]ATP: Hartmann Analytic, Braunschweig, Germany; T4 polynucleotide kinase: New England Biolabs, Frankfurt, Germany; William's medium E, L-glutamine and DMEM: Biochrome, Berlin, Germany; LightCycler 480 Probes Master, penicillin-streptomycin, poly(dI-dC), cOmplete EDTA free protease inhibitor cocktail, PhosSTOP phosphatase inhibitor cocktail, 0.45 µm PVDF membrane and Cell Death Detection ELISA^PLUS^ Kit: Roche, Mannheim, Germany; Quantikine Cytochrome c ELISA, recombinant IL-1β and recombinant TNFα: R&D Systems, Minneapolis, USA; RNeasy Plus Mini Kit and QuantiTect Reverse Transcription Kit: Qiagen, Hilden, Germany; primer and probes for quantitative RT-PCR 2: Sigma, Steinheim, Germany; actinomycin D (ActD): Enzo Life Sciences, Lörrach, Germany; p38 inhibitor RN3503 and JNK inhibitor SP600125: Prof. S. Laufer, University of Tübingen, Germany; Bradford Quick Start Dye: Bio-Rad Laboratories, Munich, Germany; ECL western blotting detection reagents, GE Healthcare, Little Chalfont, UK; pJNK, JNK, PARP, XIAP, A20/TNFAIP3, α-tubulin, pro-caspase-3 and cleaved caspase-3 antibodies: Cell Signaling, Danvers, USA; horseradish peroxidase conjugated anti-rabbit and anti-mouse secondary antibodies: Jackson Immuno Research, Newmarket, UK.

### Preparation of total, cytosolic and mitochondrial extracts

For total extracts 2×10^6^ primary mouse hepatocytes were washed with PBS, detached from the plate using a scraper and collected by centrifugation at 2150×g, 4°C for 3 min. The cells were lysed in 100 µl buffer (20 mM Tris/HCl pH 7.4, 136 mM NaCl, 2 mM EDTA, 10% glycerol, 4 mM benzamidine, 50 mM β-glycerophosphate, 20 mM Na-diphosphate, 10 mM NaF, 1 mM Na_3_VO_4_, 1% Triton X-100 supplemented with the protease inhibitors 5 µg/ml aprotinin, 5 µg/ml leupeptin, 1% PhosSTOP and 10% cOmplete) by shaking at 4°C for 25 min followed by a final centrifugation at 20,800×g, 4°C for 10 min. For preparation of cytosolic and mitochondrial extracts 4×10^6^ cells were treated as described above. The cell pellet was dissolved in 140 µl buffer (10 mM HEPES-KOH, 250 mM sucrose supplemented with protease inhibitors cOmplete (1%) and phosphatase inhibitor PhosSTOP (1%)), incubated on ice for 10 min and centrifuged at 500×g, 4°C for 3 min to remove the nuclei. Crude mitochondria were obtained from the supernatant by a further centrifugation at 10,000×g for 15 min. This fraction was washed twice with H8 buffer (20 mM Tris-HCl, pH 7.5, 2 mM EDTA, 2 mM EGTA, 6 mM ß-mercaptoethanol, 1% SDS) and left shaking for 10 min at 95°C. The cytosolic fraction was ultra-centrifuged at 100,000 g to purify the extracts. All lysates were used for immunoblotting; cytosolic fractions were additionally used for the detection of cytochrome c release by ELISA.

### DEVDase assay

The activity of the executioner caspase-3/-7 was measured by the fluorogenic DEVDase assay as previously described. See also S1 Protocol.

### Viability assay

To study the viability of primary hepatocytes after the different treatments the MTT assay was used. Detailed information is given in S1 Protocol.

### Cell Death detection ELISA

To quantify the amount of DNA fragmentation in hepatocytes (1×10^6^) after treatment with the different stimuli the cell death detection ELISA^PLUS^ Kit was used and performed according to the manufacture’s instruction (for detailed information see S1 Protocol).

### Immunoblotting

60–80 µg of total protein was separated by SDS-PAGE (10% or 12.5%) and transferred to a 0.45 µm pore size PVDF membrane. The membrane was blocked for 1 h in 5% milk TBST and subsequently probed with primary antibody solution over night at 4°C. JNK, phospho-JNK, A20/TNFAIP3, PARP, cleaved caspase-3 and α-tubulin antibodies were used at 1∶1000, XIAP at 1∶500 and pro-caspase-3 at 1∶2500. Afterwards the membrane was incubated with the respective horseradish peroxidase-labeled secondary antibody for 1 h at room temperature. The signal was visualized by enhanced chemiluminescence reagents.

### Cytochrome c ELISA

The amount of cytochrome c in cytosolic extracts was determined using an ELISA kit from R&D. Cytosolic fractions were obtained as described above. The ELISA was performed according to the manufacturer’s instruction. Briefly, the extracts were diluted 1∶50 in calibrator diluent. Samples, standards and positive control were transferred to the microplate, the conjugate was added and incubated for 2 h at room temperature. The plate was washed and incubated with the substrate solution for 30 min. The reaction was stopped and the absorbance was measured at 405 nm. Cytochrome c concentrations were calculated using the standard concentration curve.

### Electrophoretic mobility shift assay (EMSA)

Nuclear extracts were prepared and the electrophoretic mobility shift assay was performed according to protocols given in S1 Protocol.

### RNA isolation, cDNA synthesis and qRT-PCR

Total RNA was isolated using the RNeasyPlus Kit of Qiagen, according to the manufacturer’s directions. The quantity and purity of RNA was determined by measuring the optical density at 260 and 280 nm. 600 ng total RNA was reverse transcribed to cDNA with TaqMan Reverse Transcription Reagents (Applera GmbH, Darmstadt, Germany). For qRT-PCR we used the Fluidigm's Biomark high throughput qPCR chip platform (Fluidigm Corporation, San Francisco, CA, USA) with pre-designed gene expression assays from Applied Biosystems according to the manufacturer’s instructions [16]. Data were analyzed using the ddCt method and expression values were normalized to the expression levels of the β-actin gene.

### Mathematical modeling and simulation

The model is based on ordinary differential equations and mass action kinetics and was implemented using Copasi [17]. It comprises 53 species, 69 reactions and 70 parameters. Model equations can be found in S1 Protocol and a list of model species in S1 Table. 58 parameter values were taken from literature [14], [18] and five parameters were adjusted to published data, namely the parameters for formation of the IL-1β receptor complexes (S1 Fig.) were adopted to Western Blot data from Jiang et al. 2002 [7]. Seven parameters were adapted to the experimental data presented in this study as explained in more detail in S1 Protocol. Shortly, two parameters (k_v12_ and k_v13_) were adjusted so that the time course of phosphorylated JNK (S2 Fig.) matches the respective immunoblot data with. The remaining five parameters were adjusted to reproduce the measured caspase-3 activity in all experimental settings. A list of parameters with the respective reference can be found in S2 Table.

### Statistical analysis

Values are expressed as means ± standard deviation (s.d.). Statistical analyses of data sets were performed by using One-way ANOVA followed by Bonferroni’s post hoc test. P-values were calculated and p<0.05 was considered as significant.

## Results

### IL-1β sensitizes primary mouse hepatocytes to FasL-induced caspase-3/-7 activation but partially protects them from apoptosis

**Figure 1:**
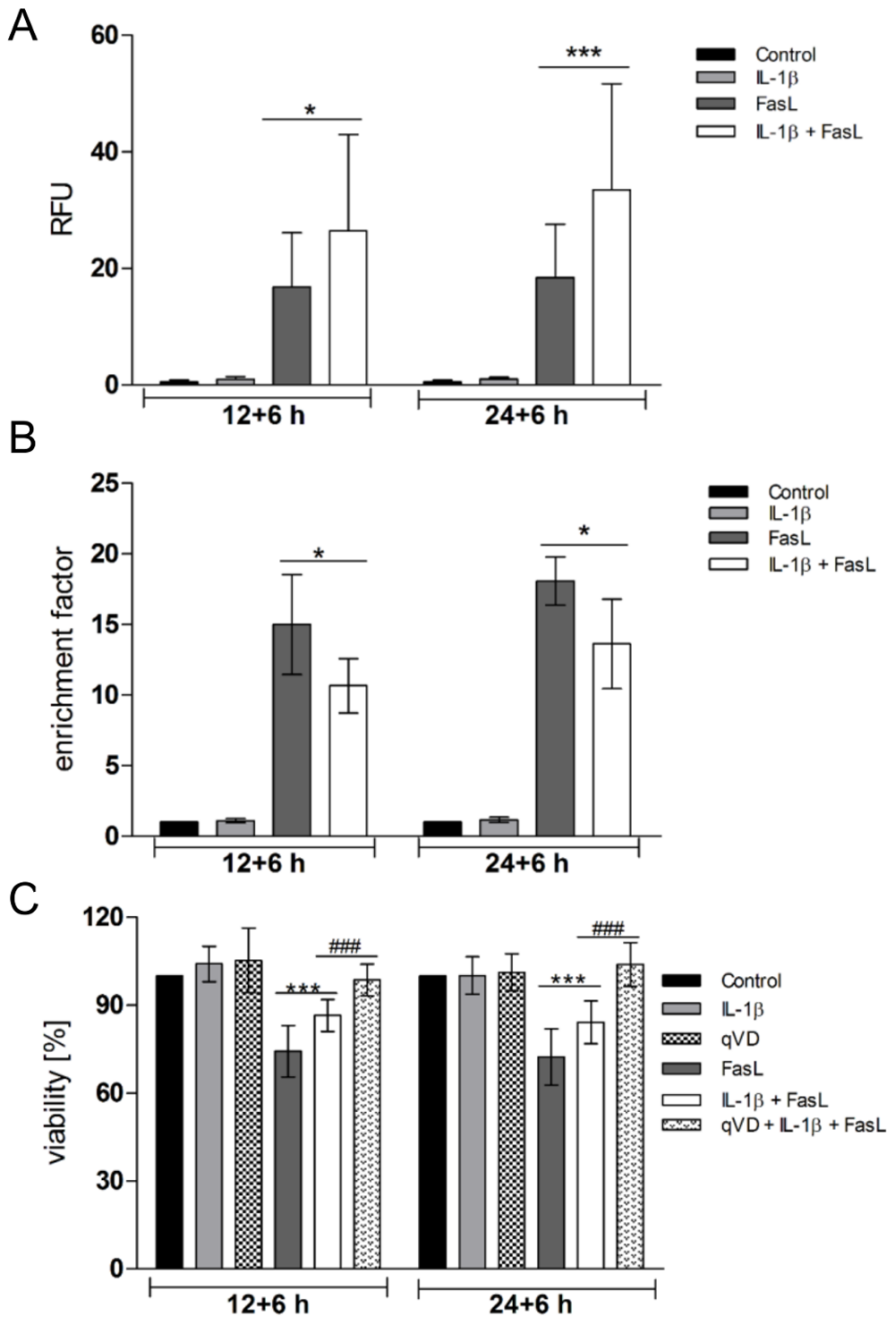
IL-1β sensitizes primary murine hepatocytes to FasL-induced caspase-3/-7 activity despite partially protecting from apoptosis. (A) Caspase-3/-7 activity determined by a fluorogenic DEVDase assay of cells treated with IL-1β (20 ng/ml), FasL (50 ng/ml), or IL-1β + FasL for the indicated times. Cells were pre-treated with IL-1β for 12 or 24 h followed by 6 h FasL stimulation. (B) Detection of DNA-fragmentation by cell death ELISA in hepatocytes treated as described above. (C) MTT viability assay of cells treated with IL-1β, FasL or both for the indicated times, in the presence or absence of pre-incubation with 25 µM of the pan-caspase inhibitor Q-VD-OPh 30 min prior to cytokine stimulation. Values are referred to untreated controls and represent 12 independent experiments ± s.d. except for treatment with IL-1β n = 4 (*p<0.05, ***p<0.001, IL-1β + FasL versus FasL-treated cells, ^###^p<0.01, IL-1β + FasL versus QVD + IL-1β + FasL).

**Figure 2:**
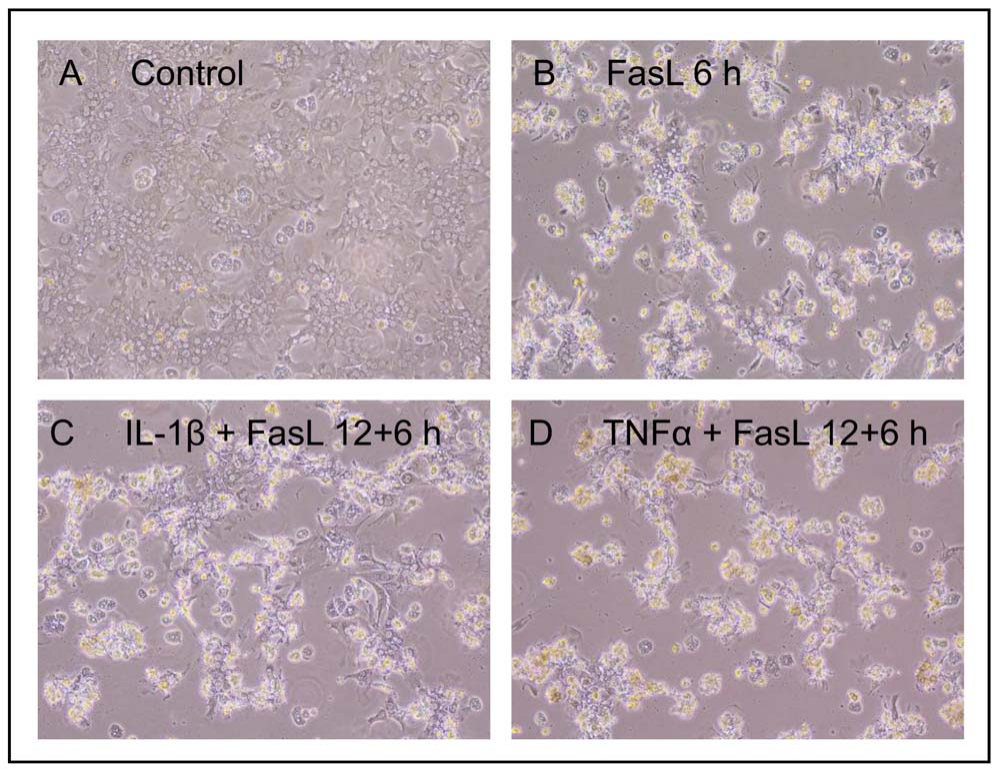
While TNFα sensitizes primary hepatocytes to FasL-induced cell death, IL-1β partially protects from this process. Phase contrast microscopic analysis of hepatocytes that were untreated (A) or treated with FasL (50 ng/ml) for 6 h (B–D), either pre-incubated with IL-1β (20 ng/ml) (C) or TNFα (25 ng/ml) (D) for 12 h (original magnification, 100 x).

**Figure 3:**
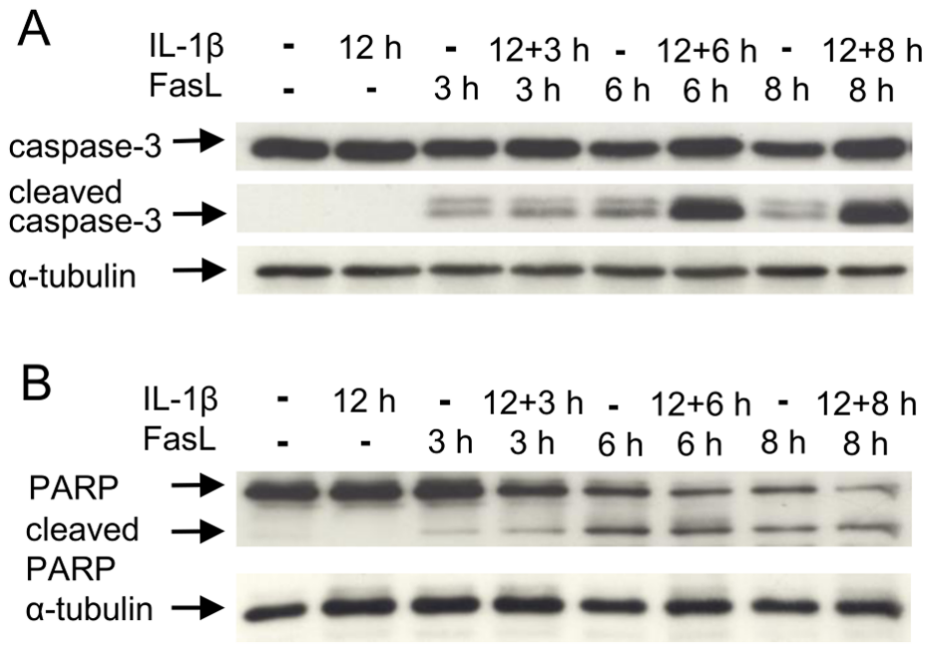
IL-1β sensitizes primary murine hepatocytes to FasL-induced caspase-3 activation, but not to increased PARP cleavage. (A) Anti-active caspase-3 and (B) anti-PARP immunoblotting of total cellular extracts from primary hepatocytes treated with IL-1β (20 ng/ml, 12 h) or FasL (50 ng/ml, 3, 6, or 8 h) alone or in combination (12+3 h, 12+6 h or 12+8 h). As a loading control membranes were reprobed with an antibody detecting α-tubulin. Representative immunoblots are shown, n = 3.

We previously reported a sensitizing effect of TNFα on FasL-induced caspase-3/-7 activation and apoptosis and identified the underlying mechanism [2]. Here we studied the influence of the pro-inflammatory cytokine IL-1β on collagen-cultured primary murine hepatocytes stimulated with FasL. Cells were pre-treated with 20 ng/ml IL-1β for 12 or 24 h followed by incubation with 50 ng/ml FasL for another 6 h. Similar to the TNFα/FasL sensitization, we noted a clear increase in caspase-3/-7 activity in cells treated with IL-1β and FasL as compared to those treated with FasL alone (Fig. 1A). A pre-incubation of at least three hours was needed and the dose of IL-1β can be reduced to 1 ng/ml to preserve the sensitizing effect on FasL-induced caspase-3/-7 activation (S3A and B Figs.). Surprisingly, however, whereas the combined treatment of TNFα + FasL enhanced cell death [2], we noticed a significant reduction in cell death upon IL-1β + FasL incubation. Two different cell death assays, MTT (Fig. 1C) and an ELISA, which measures DNA fragmentation (Fig. 1B) independently revealed that IL-1β was capable of diminishing FasL-induced cell death despite triggering a higher caspase-3/-7 activity (Fig. 1A). The cell death was clearly caspase-dependent as it was reduced in the presence of the pan-caspase inhibitor Q-VD-OPh (Fig. 1C). We confirmed this paradoxical finding by microscopic analysis (Fig. 2). Hepatocytes showed typical hallmarks of apoptosis like cell shrinkage, plasma membrane blebbing and subsequent cell detachment after 6 h of FasL incubation (Fig. 2B). Pre-incubation with IL-1β followed by FasL treatment reduced all these apoptotic features as compared to FasL treatment alone (Fig. 2C), whereas pre-treatment with TNFα enhanced them (Fig. 2D). To confirm that caspase-3 activity was indeed enhanced by IL-1β sensitization, we monitored caspase-3 processing by immunoblotting. As shown in Fig. 3A, the cleavage of caspase-3 into its active p17 fragment was significantly increased after treating hepatocytes with IL-1β for 12 h plus FasL for 3, 6, or 8 h as compared to a single FasL treatment. Since increased caspase-3 activity and processing did not translate into diminished, but rather enhanced viability, we examined the cleavage of PARP, a typical caspase-3 target substrate involved in apoptosis induction. As shown in Fig. 3B PARP was similarly cleaved after FasL treatment irrespective of whether IL-1β was pre-incubated or not. By contrast, co-stimulation of cells with TNFα + FasL showed increased levels of cleaved PARP when compared to treatment with FasL alone (S4 Fig.).

### IL-1β sensitization of FasL-induced caspase-3/-7 activation proceeds via JNK1/2

**Figure 4:**
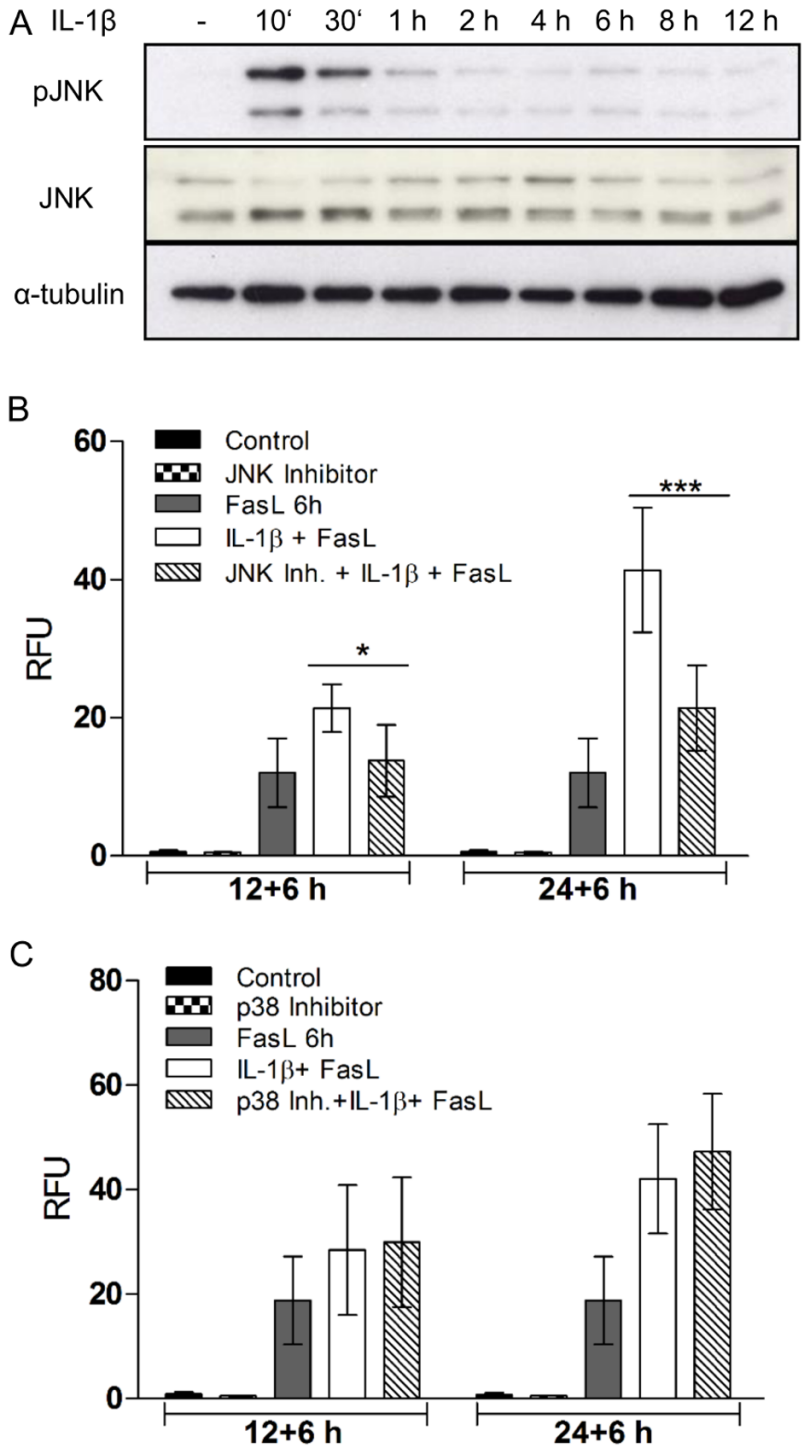
JNK1/2 but not p38 are involved in the sensitization of FasL-induced caspase-3/-7 activation by IL-1β. (A) JNK1/2 phosphorylation determined by anti-pJNK1/2 immunblot analysis after treatment with IL-1β (20 ng/ml) for the indicated times (10 min, 30 min, 1 h, 2 h, 4 h, 6 h, 8 h and 12 h). Total JNK1/2 and α-tubulin were used as loading controls. One representative experiment is shown. (B) Caspase-3/-7 activity measured by fluorogenic DEVDase assay in response to IL-1β and FasL with or without 20 µM of the JNK inhibitor SP600125 (B) or 10 µM of the p38 inhibitor RN3503 (C) for 12 and 6 h or 24 and 6 h. Means of 6 independent experiments ± s.d. are shown except for treatment with the inhibitors (n = 3) (*p<0.05, ***p<0.001, IL-1β + FasL versus SP600125 + IL-1β + FasL-treated cells).

We showed that JNK1/2 are crucial mediators of the sensitizing effect of TNFα on FasL-induced apoptosis [2]. These kinases are involved in the degradation of anti-apoptotic Bcl-2 proteins and the induction of type II apoptosis [19]. We performed anti-phospho JNK1/2 immunoblotting on total cellular extracts of primary hepatocytes to determine the phosphorylation status of JNK1/2 after treatment with IL-1β (Fig. 4A). IL-1β triggered rapid phosphorylation of JNK1 and JNK2 (double band) within the first 30 min of incubation. After 1 h this phosphorylation was reduced, but remained at a basal level for at least 18 h.

To study if increased JNK1/2 phosphorylation/activation by IL-1β was required for the sensitization effect on caspase-3/-7 activity we treated primary hepatocytes with 20 µM of the JNK inhibitor SP600125 for 30 min before IL-1β addition. JNK inhibition effectively reduced the sensitizing effect of IL-1β on FasL-induced caspase-3/-7 activity to the same level as with FasL treatment alone (Fig. 4B). In contrast, the p38 mitogen-activated protein kinase (MAPK), which is also activated by IL-1β [20], is not involved in the IL-1β-mediated increase of active caspase-3/-7 levels since pre-incubation with the p38 MAPK inhibitor RN3503 (10 µM) did not show any effect on the IL-1β + FasL-induced DEVDase activity (Fig. 4C).

### Bim and Bid but not XIAP are crucial mediators of the sensitization effect of IL-1β on FasL-induced caspase-3/-7 activation

**Figure 5:**
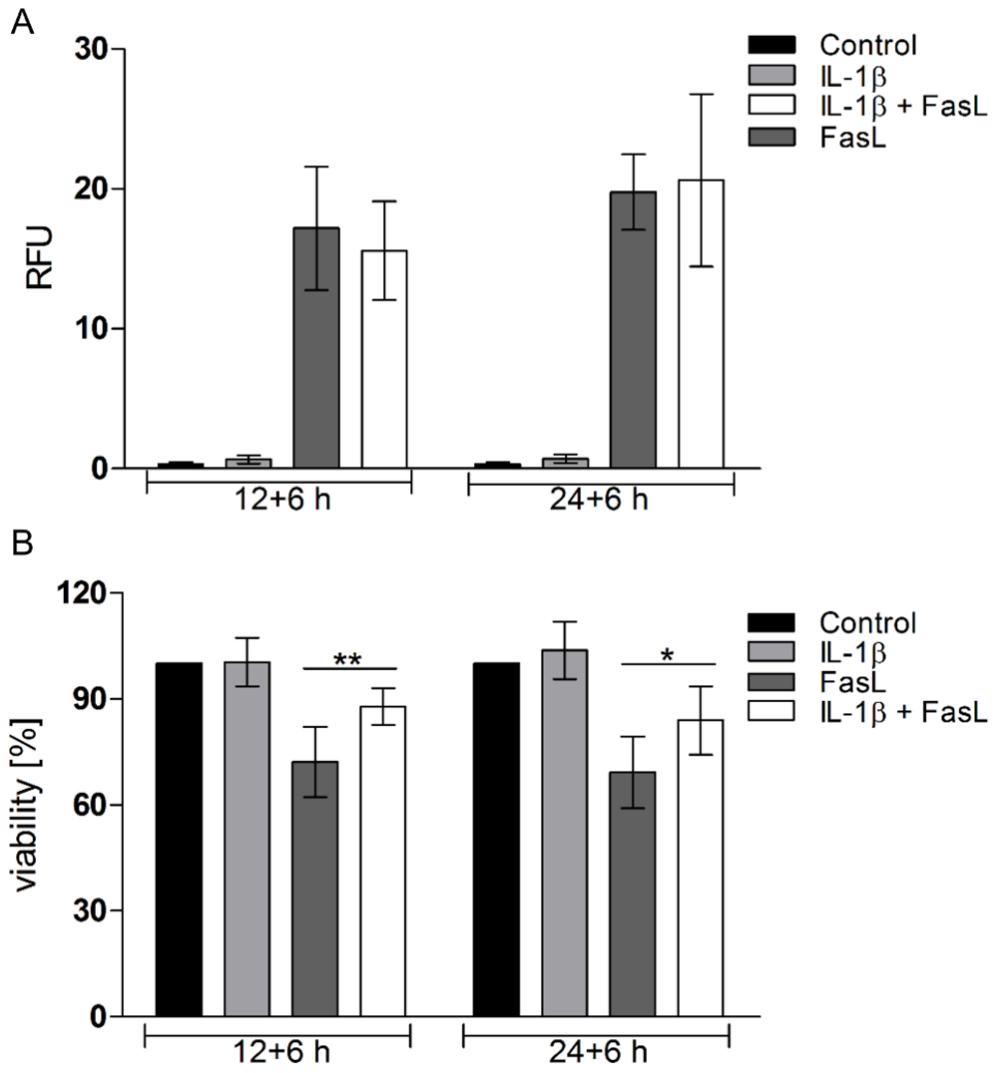
Bim deficiency influences FasL-induced caspase-3/-7 activity, but not cell viability in IL-1β pre-treated hepatocytes. Hepatocytes from Bim^-/-^ were pre-treated with IL-1β for 12 or 24 h followed by 6 h FasL incubation. Caspase-3/-7 activity (A) and cell viability (B) were determined by the DEVDase and the MTT assay. Means of 6 independent experiments ± s.d. are shown except for treatment with IL-1β (n = 3) (*p<0.05, **p<0.01, FasL versus IL-1β + FasL, values for MTT assay are referred to untreated control).

Further studies were performed to unravel the signaling pathway leading to the sensitization effect of IL-1β on FasL-induced caspase-3/-7 activation. We first focused on the BH3-only protein Bim because it was shown to be phosphorylated by activated JNK [21]. JNK-mediated Bim phosphorylation enhances the pro-apoptotic activity of Bim towards Bax/Bak-mediated MOMP and subsequent cytochrome c release, a pivotal event for type II mediated apoptosis [22]–[24]. We therefore isolated primary hepatocytes from Bim^-/-^ mice and treated them with IL-1β, FasL or both. The increase in FasL-induced caspase-3/-7 activation by IL-1β (Fig. 1A) was entirely abolished in Bim^-/-^ hepatocytes (Fig. 5A), whereas the protective effect of IL-1β on cell viability was conserved (Fig. 5B). This indicates that Bim was crucial for enhanced caspase-3/-7 activation in response to IL-1β (Fig. 5A). As previously published by using siRNA knockdown strategies, Bim^-/-^ hepatocytes did also not show any increased caspase-3 activation or cell death in response to TNFα + FasL (S5A and B Figs.) [2]. Our data therefore underline the pivotal role of Bim in caspase-3/-7 activation in response to both TNFα and IL-1β.

**Figure 6:**
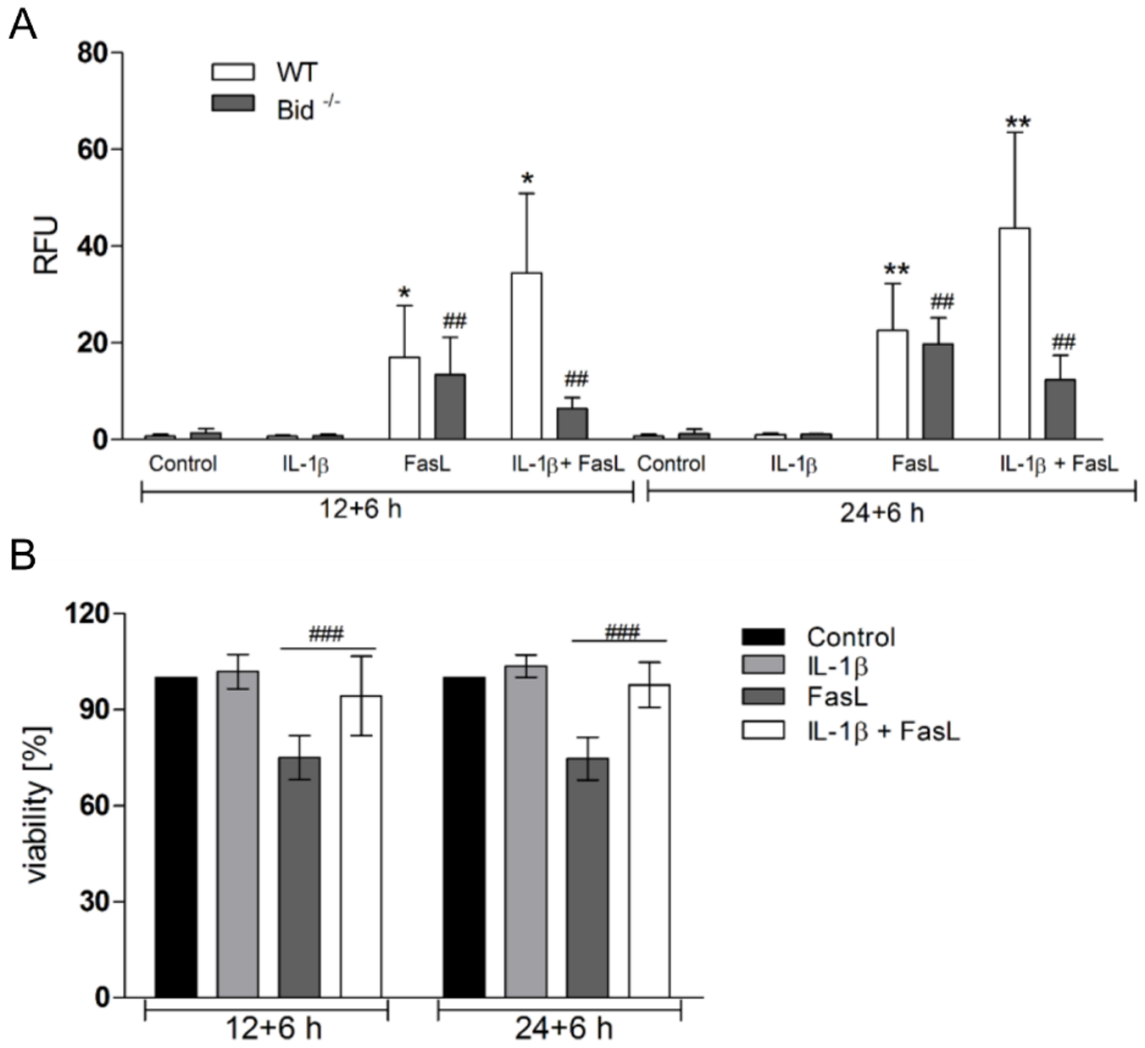
Bid is involved in the sensitizing effect of IL-1β on FasL-induced caspase-3/-7 activation. (A) Caspase-3/-7 activity in wt and Bid^-/-^ hepatocytes after pre-treatment with IL-1β for 2 h or 24 h alone or followed by FasL for 6 h as well as single FasL stimulation for 6 h. (B) MTT cell viability assay in Bid^-/-^ hepatocytes treated as described above. Means of 6 independent experiments ± s.d. are shown except for treatment with IL-1β (n = 4). *p<0.05, ***p<0.001: IL-1β + FasL versus FasL treatment in wt cells; ^##^p<0.01, ^###^p<0.001: IL-1β + FasL versus FasL treatment in Bid^-/-^ cells. Values for MTT assay are referred to untreated control.

Bid is another BH3-only protein involved in the crosstalk between TNFα and FasL because TNFα-sensitization on FasL-induced caspase-3/-7 activation and apoptosis was abolished in Bid^-/-^ cells [2]. When Bid^-/-^ hepatocytes were pre-treated with IL-1β followed by FasL stimulation the increased caspase-3/-7 activity was abolished, indicating that Bid was also necessary for the sensitization by IL-1β (Fig. 6A). Interestingly, the amount of active caspase-3/-7 was even reduced below the levels of FasL stimulation alone, emphasizing the protective effect mediated by IL-1β. Consequently, when cell viability was measured in IL-1β + FasL-treated Bid^-/-^ hepatocytes by MTT assay, it almost reached the level of untreated cells (Fig. 6B). Importantly, caspase-3/-7 activation was comparable between FasL-stimulated wt and Bid^-/-^ cells, indicating that Bid was not required for FasL-induced apoptosis (as shown in [25]) but was crucial for the IL-1β sensitizing effect in primary hepatocytes cultured on collagen monolayers.

**Figure 7:**
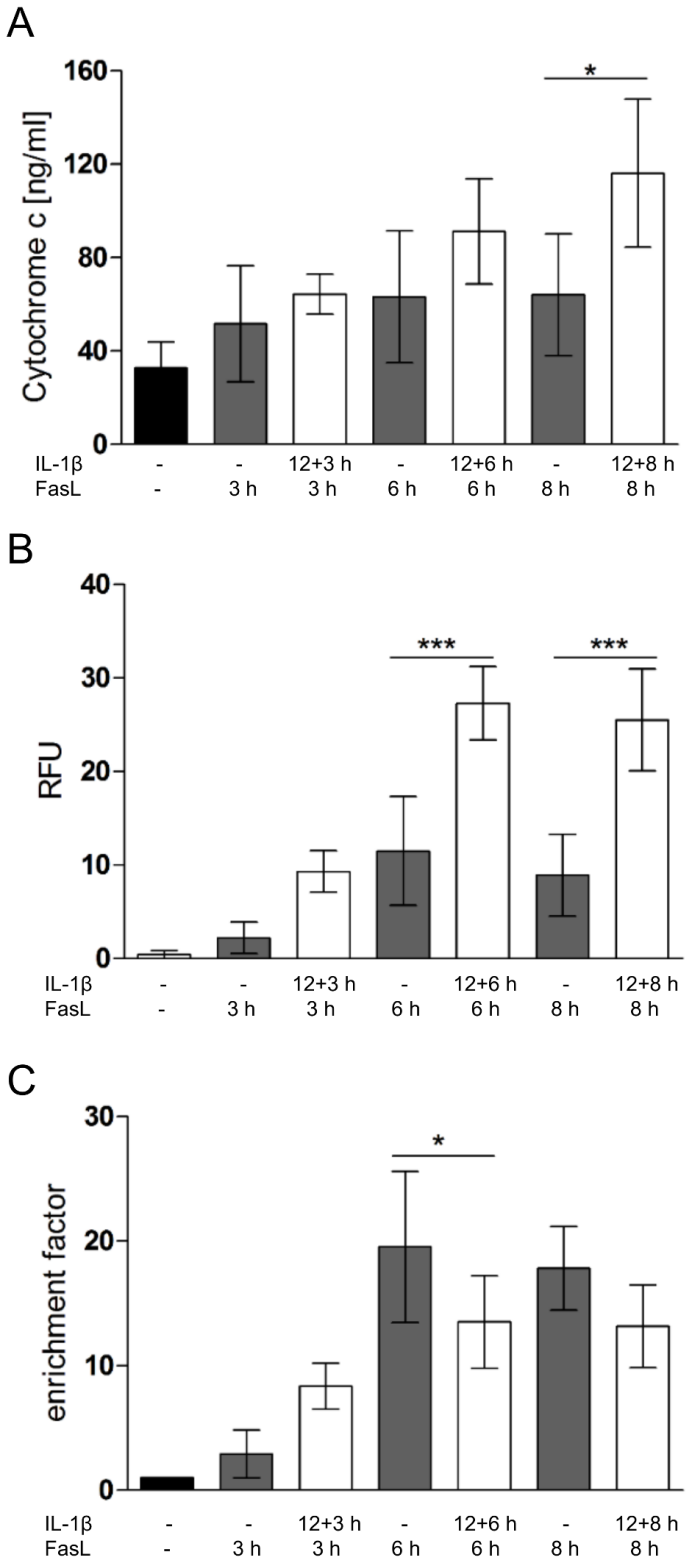
Time-dependent cytochrome c release, caspase-3/-7 activity and DNA fragmentation in hepatocytes treated with IL-1β and FasL. Primary hepatocytes were treated with FasL or IL-1β + FasL for the indicated times before performing cytochrome c release by ELISA (A), a fluorogenic DEVDase caspase assay (B), or a cell death DNA fragmentation assay (C). Values for the DNA fragmentation assay are referred to the untreated control. Five independent experiments ± s.d. are displayed except for the incubation time 12+3 h (n = 4) (*p<0.05, ***p<0.001 IL-1β + FasL versus FasL treated cells at the corresponding time point).

Bim and Bid are known to activate Bax/Bak-mediated MOMP. Thus, if both proteins are involved in the IL-1β sensitization effect on FasL-induced caspase-3 activation, they are likely to do so via triggering the release of cytochrome c. We therefore measured cytochrome c release by an ELISA assay and compared it to caspase-3/-7 activation (DEVDase assay) and DNA fragmentation (Fig. 7). While FasL alone did not much trigger cytochrome c release, the pre-treatment with IL-1β (Fig. 7A) or TNFα (S6A Fig.) enhanced this process, especially at 12+8 h treatments. Caspase-3/-7 activity levels increased accordingly after the combined IL-1β + FasL and TNFα + FasL as compared to a single FasL treatment (Fig. 7B, S6B Fig.). However, DNA fragmentation as an indicator of cell death was reduced by IL-1β after 6 and 8 h but not after 3 h of FasL treatment, indicating that cytoprotection by IL-1β is a delayed process and probably involves transcriptional activation in contrast to caspase-3/-7 sensitization (Fig. 7C). By contrast, as reported before, TNFα in combination with FasL (3–8 h) showed increased cell death as compared to FasL treatment alone (S6C Fig.).

Caspase-3/-7 and also caspase-9 activities are regulated by the endogenous inhibitor XIAP [12]. We therefore tested XIAP^-/-^ cells for the sensitizing effect of IL-1β on FasL (10–25 ng/ml). As expected and previously reported (Walter et al., 2006), XIAP-deficient primary hepatocytes exerted a higher level of caspase-3/-7 activity in response to FasL than wt cells (S7A and B Figs.). At 50 ng/ml FasL, IL-1β did not further increase caspase-3/-7 activity as compared to FasL alone (S7A Fig.). However at 10 and 25 ng/ml FasL, we clearly detected a caspase-3/-7-sensitization effect of IL-1β in XIAP^-/-^ cells (S7B Fig.). To further test whether IL-1β affects XIAP protein levels in wild type hepatocytes and thus might contribute to increased hepatocyte survival upon IL-1β pre-treatment, endogenous XIAP protein levels were analyzed by immunoblotting (S7C Fig.). However, XIAP protein levels did not change in the presence of FasL or IL-1β. Thus, our data indicate that IL-1β sensitization of FasL-induced caspase-3/-7 activation is independent of XIAP.

### Pre-incubation of FasL-treated hepatocytes with IL-1β or TNFα differentially affects NF-κB activation

**Figure 8:**
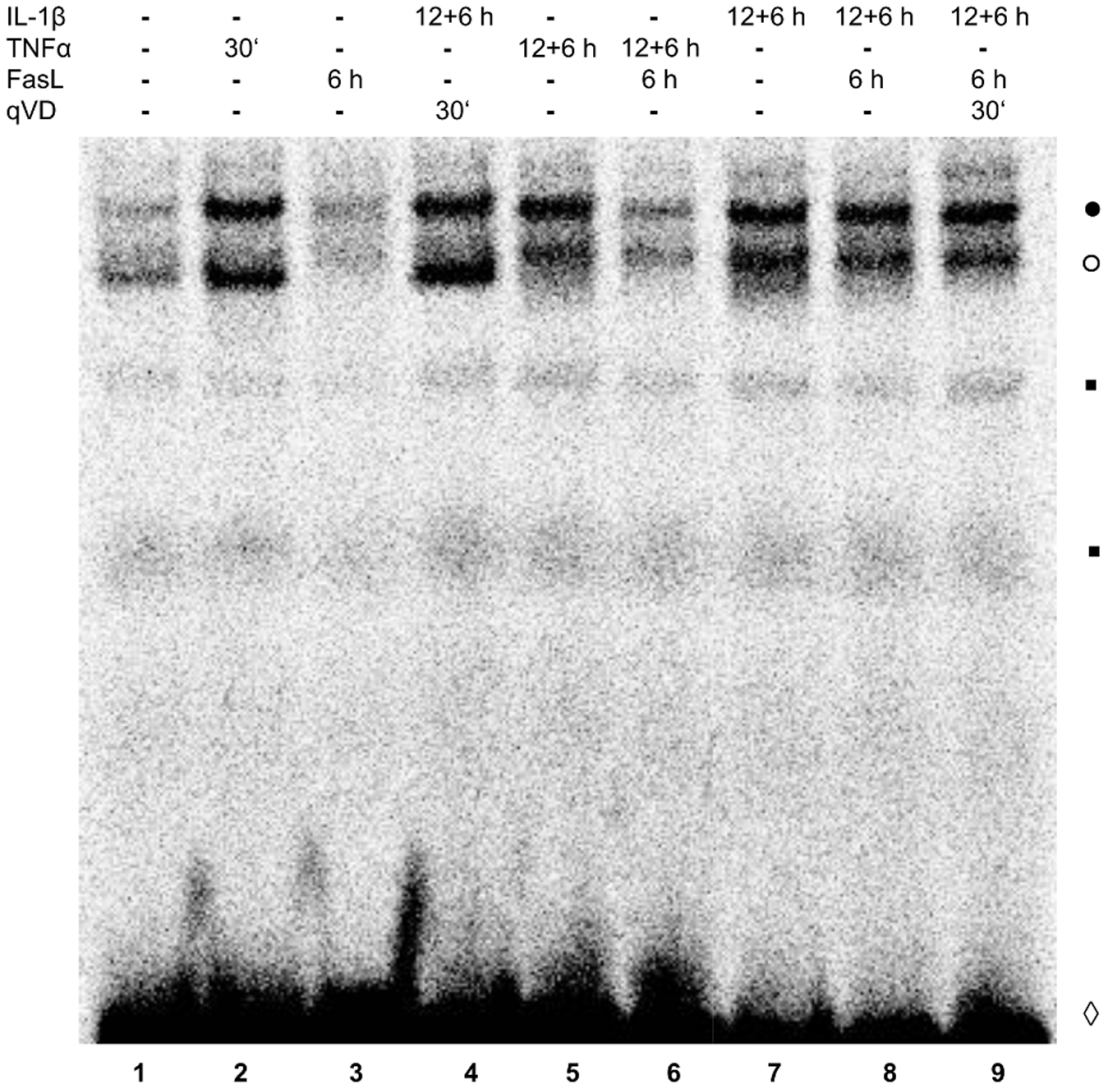
NF-κB activation is decreased after combinatorial stimulation with TNFα + FasL, but persists after IL-1β + FasL stimulation. Nuclear extracts of primary hepatocytes were prepared after treatment with IL-1β (20 ng/ml), TNFα (25 ng/ml), and FasL (50 ng/ml) for the indicated times. Additionally, cells were pre-treated for 30 min with the pan-caspase inhibitor Q-VD-OPh (20 µM) followed by IL-1β or IL-1β + FasL stimulation. Control cells remained untreated (lane 1). Equal nuclear protein amounts were analyzed for NF-κB DNA binding activity in an EMSA.•○ NF-κB-DNA complexes, ▪ non-specific binding to the probe, ◊ unbound oligonucleotide. The result of the EMSA was reproduced and one representative EMSA is shown.

Both, IL-1β and TNFα trigger the activation of the transcription factor NF-κB, which mediates induction of anti-apoptotic genes [13], [26]. To compare the impact of IL-1β and TNFα on NF-κB activation in the presence or absence of FasL, primary hepatocytes were pre-treated with either cytokine for 18 h alone or for 12 h followed by FasL stimulation for further 6 h. Nuclear extracts were prepared and NF-κB DNA binding was analysed by electrophoretic mobility shift assays (EMSA). Treatment of hepatocytes by IL-1β or TNFα for 18 h resulted in a strong activation of NF-κB DNA binding (Fig. 8, lane 5, 7). By contrast, while NF-κB activity decreased after combinatorial stimulation with TNFα + FasL, this was not the case after IL-1β + FasL. To study whether IL-1β induced NF-κB activation is influenced by caspase-3/-7 activity, hepatocytes were pre-treated with the pan-caspase inhibitor Q-VD-OPh before treatment with IL-1β and FasL. NF-κB activity was not impaired by caspase inhibition (Fig. 8, lane 9).

**Figure 9:**
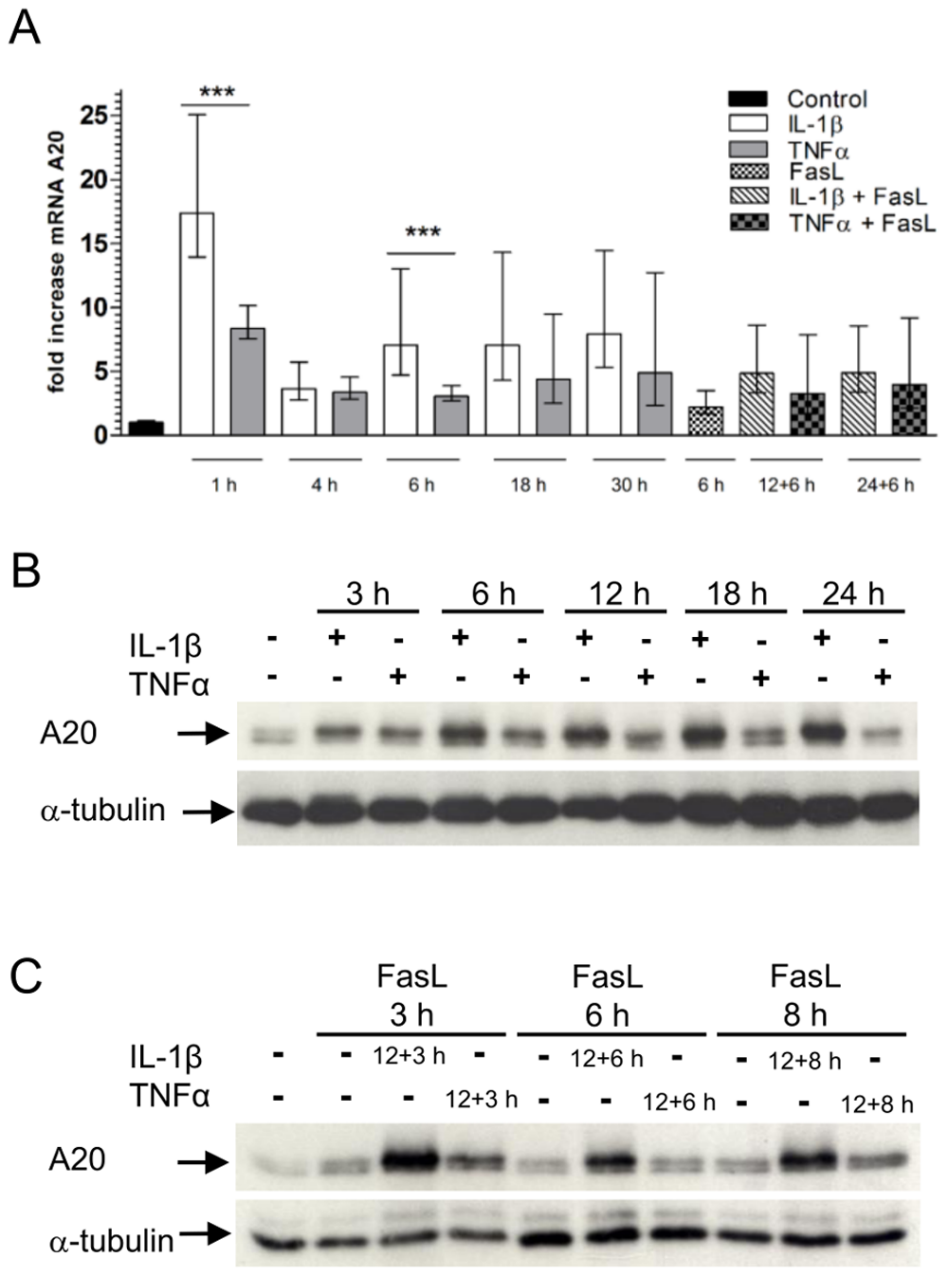
Induction of A20 upon IL-1β or TNFα treatment. (A) mRNA of primary hepatocytes treated with IL-1β (20 ng/ml), TNFα (25 ng/ml), FasL (50 ng/ml) or the combination was isolated after the indicated time. The gene expression of A20 was analysed by qRT-PCR. Four independent experiments ± s.d. are displayed. (***p<0.001, IL-1β versus TNFα treated cells at the corresponding time point). (B/C) A20 protein levels in wt hepatocytes treated with 20 ng/ml IL-1β, 25 ng/ml TNFα, 50 ng/ml FasL or the combination is shown for the indicated times. α-tubulin was used as a loading control. A representative immunoblot is shown (n = 3).

The ubiquitin-editing enzyme A20 is transcriptionally regulated by NF-κB [27] and acts as an inhibitor of caspase-8 [28]. We therefore determined A20 mRNA levels and found that A20 transcription increased about two fold after 1 h of IL-1β treatment as compared to TNFα stimulation (Fig. 9A). After 4 h A20 mRNA levels decreased for both cytokines but they were elevated again for IL-1β at later time points. Stimulation of the cells with FasL had no significant effect. Combinatorial stimulation with IL-1β + FasL slightly increased A20 mRNA as compared to TNFα + FasL. Likewise, A20 protein levels increased in the presence or absence of FasL upon IL-1β treatment, whereas FasL alone had no pronounced effect on A20 translation (Fig. 9B/C). TNFα also induced A20 protein synthesis, but to a lower extent compared to IL-1β treatment (Fig. 9B). Given the anti-apoptotic function of A20 [28] these data suggest that A20 may play a role in the protective effect of IL-1β on FasL-induced apoptosis.

Additionally, further target genes of NF-κB were analyzed, including cIAP1, cIAP2, cFLIP_S_ or cFLIP_L_. However, no significant difference in the expression of the corresponding genes was observed, when comparing IL-1β and TNFα treated hepatocytes (data not shown). This indicates that these genes are most probably not involved in cytoprotection mediated by IL-1β.

### Mathematical modeling confirms the mechanism of IL-1β-induced sensitization

**Figure 10:**
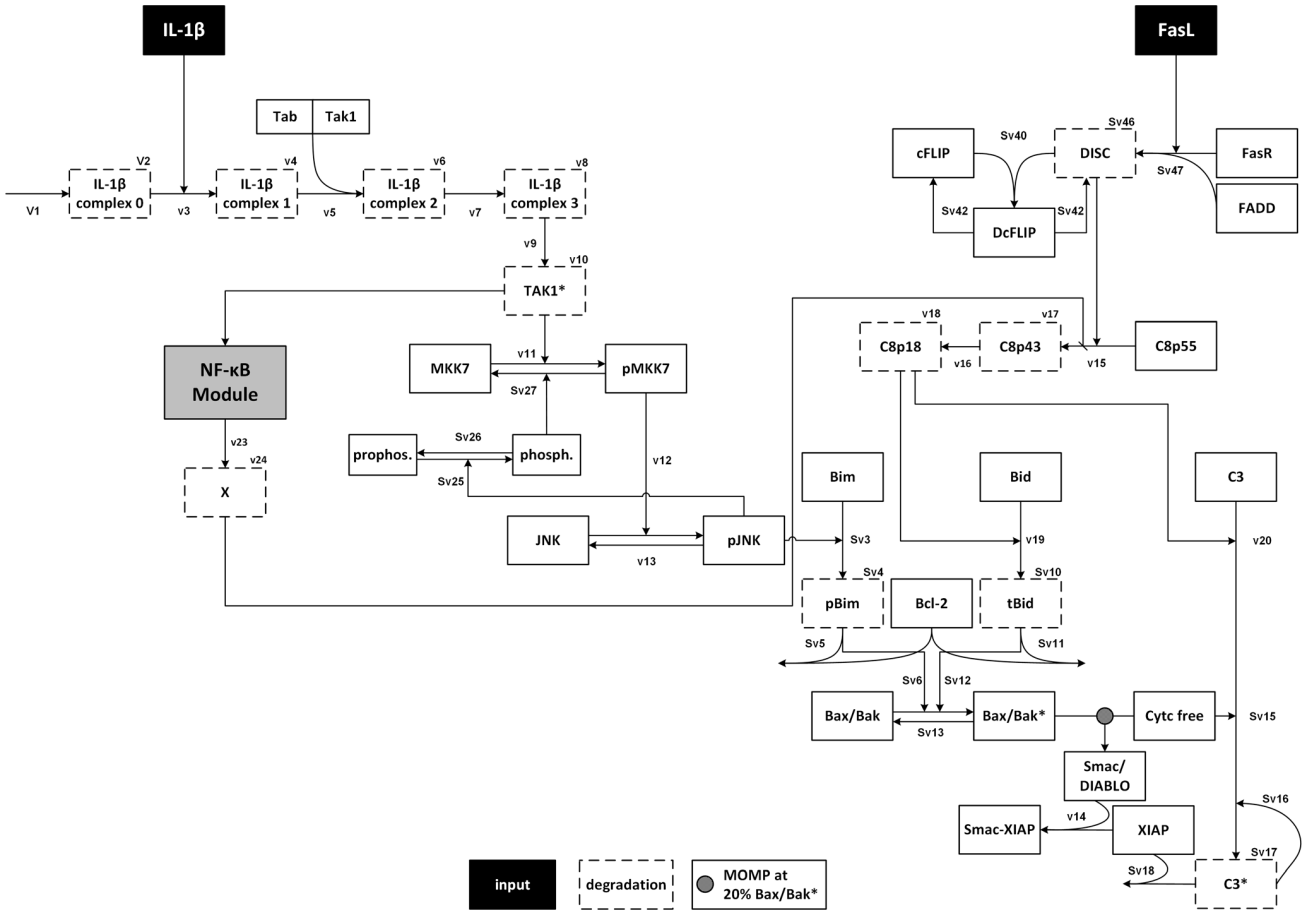
Structure of the IL-1β/FasL model. Inputs of the model are IL-1β and FasL depicted in black and model output is activated caspase-3 (C3*). The model is based on ordinary differential equation (ODE) and mass action kinetics. The integrated NF-κB model from Lipniacki et al. (Lipnicaki et al., 2004) is summarized as a gray box for clarity; the detailed scheme is depicted in S8 Fig. Degradation of species is indicated by boxes with dashed border.

**Figure 11:**
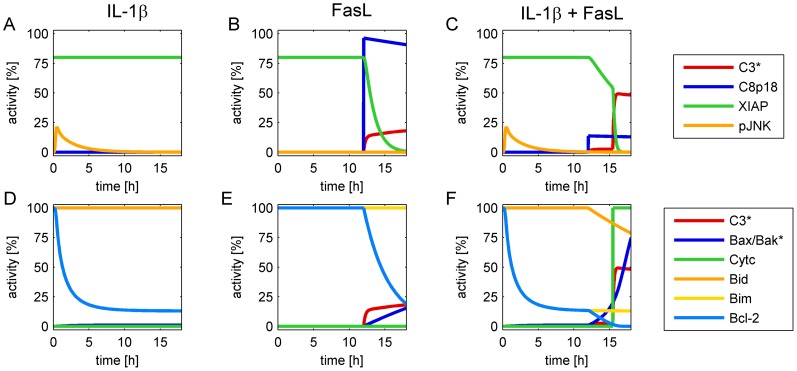
Simulation of caspase-8 and -3 activities, cytochrome c release and levels of pJNK, Bid, Bim, Bcl-2 and XIAP after treating wt hepatocytes with IL-1β, FasL or a combination of both. Simulation results for wt cells after stimulation with IL-1β (A, D), FasL (B, E) or both (C, F). IL-1β stimulus is given at time point 0, FasL is added after 12 h. Abbreviations: C8p18, active caspase-8; C3*, active caspase-3; pJNK, phosphorylated JNK.

We used an ordinary differential equation model in order to explain our experimental findings. The model was adopted from the TNFα/FasL model by Schlatter et al. [14] that already contains an NF-κB module from Lipniacki et al. [18] (S8 Fig.) and complemented with a module describing IL-1β signaling as explained in S1 Protocol. Furthermore, to model the IL-1β-induced protective effect a yet unidentified protein X was added, which possibly corresponds to A20 but this hypothesis needs further verification. The structure of the model is given in Fig. 10, and the simulations of wt hepatocytes after treatment with IL-1β, FasL, and in combination are displayed in Fig. 11. According to this model, a cellular treatment with IL-1β alone does not lead to caspase-8 and -3 activation and therefore no XIAP is consumed (Fig. 11A). IL-1β stimulation induces transient phosphorylation of JNK that in turn phosphorylates Bim leading to a consumption of Bcl-2 (Fig. 11D). The Bcl-2 buffer decreases with time and Bax/Bak gets activated, but the critical threshold of 20% Bax/Bak* is not reached. Therefore, MOMP and cytochrome c release do not occur. On the other hand, FasL stimulation induces the apoptotic pathway by activation of caspase-8. This protease directly cleaves and activates caspase-3, which is partially buffered by XIAP and therefore only reaches a weak activation level (Fig. 11B). At the same time active caspase-8 cleaves Bid into tBid, which is subsequently buffered by Bcl-2 (Fig. 11E). Bax/Bak is slightly activated but again does not reach the critical threshold; hence cytochrome c is not released into the cytosol. Pre-incubation with IL-1β for 12 h leads to JNK activation and Bim phosphorylation and again pBim is mostly sequestered by Bcl-2 proteins. However, tBid, which is subsequently generated by FasL-induced caspase-8 activation cannot be sequestered by Bcl-2 proteins anymore and thus leads to a stronger Bax/Bak activation and the release of cytochrome c into the cytosol. This release triggers a fast and strong activation of caspase-3 that reaches a significantly higher level than in cells treated with FasL alone (Fig. 11C and F).

**Figure 12:**
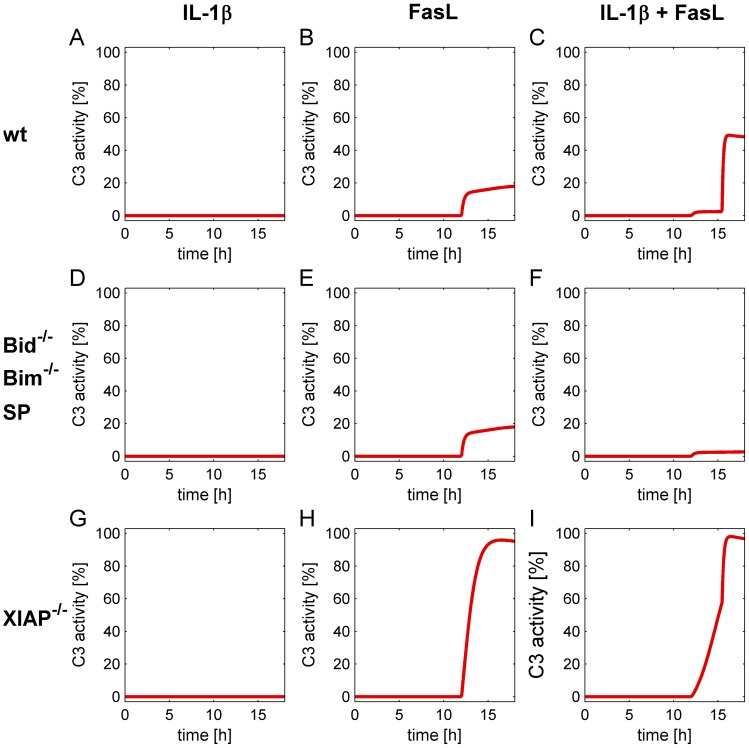
Simulation of caspase-3 activation in wt and knockout hepatocytes. (A-C) Simulation results for wt cells treated with IL-1β (A), FasL (B) or both (C). (D-F) Simulation results for Bid^-/-^ and Bim^-/-^ hepatocytes as well as wt cells treated with the JNK inhibitor SP600125, stimulation with IL-1β (D), FasL (E) or IL-1β + FasL (F). (G-I) Simulation results for XIAP^-/-^ cells treated with IL-1β (G), FasL (H) or both (I).

We then modeled the effects of Bid, Bim and XIAP deficiencies as well as JNK inhibition on caspase-3 activation (Fig. 12). Knockouts were simulated by setting the initial condition of either Bid, Bim, or XIAP to zero. JNK inhibitor treatments were simulated by setting the parameters of the reactions triggered by activated JNK (pJNK) to zero, namely k_Sv3_ and k_Sv25_. Again, IL-1β treatment alone does not activate caspase-3 in all of these scenarios (Fig. 12A,D, and G). In wt cells, stimulation with FasL leads to moderate caspase-3 activation via the type I apoptotic pathway (Fig. 12B), whereas the co-stimulation with IL-1β and FasL enhances caspase-3 activation (Fig. 12C). The kinetics of caspase-3 activation are equal in Bid^-/-^ and Bim^-/-^ hepatocytes as well as in wt cells treated with the JNK inhibitor (Fig. 12D-F), although the mechanisms causing an elimination of the sensitizing effect are different in each setting (as shown in the individual simulations of each knockout in S9-S12 Figs.). The IL-1β-induced caspase-3 sensitizing effect was abrogated in these mutants. Caspase-3 activity was even reduced below the level of a single FasL treatment in these knockout/inhibitor conditions, reflecting the protective effect mediated by IL-1β via induction of protein X and subsequent inhibition of caspase-8 activation (e.g. S9C Fig.). This protective effect of IL-1β on the level of caspase-3 activity can also be seen in the simulations of wt cells (Fig. 11B,C). Activity of C8p18 in response to IL-1β + FasL is lower compared to single FasL stimulation due to the inhibitory action of protein X. Accordingly, also caspase-3 activity in the first few hours following FasL stimulation is lower (Fig. 11C, 12–15 h). However, when enough Bax/Bak is activated, cytochrome c is released resulting in rapid and complete activation of caspase-3 via the apoptosome thus reaching significantly higher levels compared to FasL stimulation. In XIAP^-/-^knockout cells (Fig. 12G-I) caspase-3 activity is high even upon single FasL treatment. Simulations showed that caspase-3 activities reach similar levels upon FasL (Fig. 12H) and IL-1β + FasL (Fig. 12I). Nevertheless, the sensitization mechanism, i.e. induction of MOMP, is conserved as shown by the steeply rising caspase-3 activity. Activation of Bax/Bak and subsequent cytochrome c release also occur in XIAP^-/-^ cells (S11 Fig.) causing the different dynamics of caspase-3 activation.

Altogether, the ODE model reproduces the experimentally observed behavior of caspase-3 activation by all single and combined stimuli in wt cells as well as in Bid^-/-^, Bim^-/-^, and XIAP^-/-^ hepatocytes. These results confirm that the network structure given in Fig. 10 is able to explain the sensitizing effect and its dependency on Bid, Bim and JNK1/2.

### Influence of cell heterogeneity on cell viability

**Figure 13.**
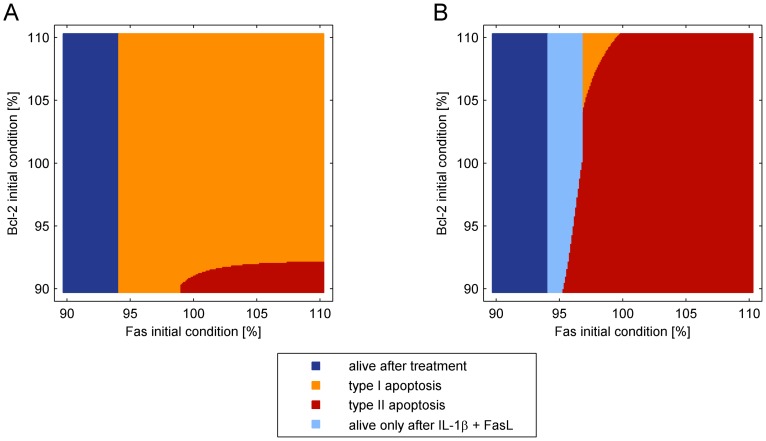
Cell fate dependent on initial levels of Fas and Bcl-2. Simulation results upon treatment with FasL (A) and IL-1β + FasL (B) for various initial levels of Fas and anti-apoptotic Bcl-2 proteins (Bcl-2) as representatives for variations at the level of DISC formation and MOMP induction, respectively. Nominal initial conditions are at 100% for both proteins and were varied ±10%. For caspase-3 levels above 1.5%, cells are classified as type I apoptotic (without cytochrome c release) or type II apoptotic (with cytochrome c release). The light blue colored fraction in (B) marks the conditions for cells that survive treatment with IL-1β + FasL, but die after single FasL stimulation (A).

An elevated viability despite increased caspase-3/-7 levels upon IL-1β + FasL co-stimulation might be due to an unequal distribution of pro- and anti-apoptotic proteins between the cells of a population. Indeed, individual cells do not equally die in response to FasL or IL-1β + FasL stimulation (Fig. 2). Accordingly, Kallenberger et al. suggested a threshold behavior in final caspase-8 activation at DISC due to a combination of intra- and interdimeric cleavage steps ensuring weak signal propagation in response to minor input concentrations and accelerated signaling and apoptosis at higher input levels [29]. This mechanism was implemented in a simplified manner by realizing a threshold concentration of DISC molecules that is necessary for the final caspase-8 activation step into the p18 fragment (C8p18). Dependence of cell fate on the initial conditions of Fas and Bcl-2 was analyzed to investigate the influence of inhomogeneous amounts of proteins among different cells (Fig. 13). Fas and Bcl-2 were chosen as representatives for variations at the level of ligand-receptor interaction and DISC formation and on the level of the Bcl-2 protein family and MOMP induction, respectively. The simulations demonstrate that most cells die via the type I apoptotic pathway in response to FasL, whereas some with lower amounts of Fas survive the treatment and a few with higher amounts of Fas and low levels of Bcl-2 die via the type II pathway (Fig. 13A). Pre-incubation with IL-1β favors cytochrome c release. Thus, the majority of cells after IL-1β + FasL treatment die via the type II pathway. Again, cells with lower amounts of Fas survive the treatment. The light blue colored fraction in Fig. 13B marks the conditions for that cells survive the treatment with IL-1β + FasL but die after single FasL stimulation (Fig. 13A) reflecting the protective effect of IL-1β on FasL-induced apoptosis via induction of the protective protein X and inhibition of caspase-8 activation. Nevertheless, in average caspase-3 activity is still higher after the combinatorial treatment compared to single FasL stimulation as demonstrated in S13 Fig. This is due to the IL-1β-induced switch in apoptotic pathways. For IL-1β + FasL stimulation, caspase-3 activity in the first hours is generally lower due to the inhibitory effects of protein X on caspase-8 and subsequently caspase-3 activity. Therefore, more cells survive the treatment and only a small fraction of cells undergoes type I apoptosis. However, these low levels of activated caspase-8 are sufficient to cause MOMP and cytochrome c release and subsequent rapid and strong caspase activation in a further large fraction of cells. This can be explained by the fact that the pool of anti-apoptotic Bcl-2 proteins was almost fully depleted during IL-1β preincubation. Altogether, the seemingly contradictory results of both increased caspase-3 activity and cell viability can be explained by taking into account a heterogeneous cell population with individual amounts of proteins.

## Discussion

IL-1β is a pro-inflammatory cytokine involved in various liver diseases [6]. In this study we report that pre-treatment of hepatocytes with IL-1β enhances FasL-induced caspase-3/-7 activity, but astonishingly diminishes FasL-induced apoptosis. In the literature, contrary effects of IL-1β on cell viability are described. An *in vivo* study demonstrated that a pre-treatment of mice with IL-1β protected the animals from subsequent liver injury by Fas-dependent apoptosis. These mice showed decreased liver enzyme serum concentrations, reduced caspase-3/-7 activity levels and prolonged survival [3]. On the other hand, in pancreatic MIA PaCa-2 cells IL-1β mediated cell death by triggering the phosphorylation of JNK due to ER stress [30].

Our results demonstrate that treatment of hepatocytes with IL-1β alone resulted in a time-dependent phosphorylation of JNK1/2, which was previously described in mouse embryonic fibroblasts and in the rabbit liver [31], [32]. This JNK1/2 activation was essential for the crosstalk of IL-1β in the presence of FasL as its inhibition, but not that of p38 MAPK, abolished the increase in caspase-3/-7 activity that was mediated by IL-1β pre-treatment. JNK is known to be crucial for modulating the activity of pro-apoptotic BH3-only proteins. *In vitro* and *in vivo* (thymus) studies showed that Bim and Bmf were phosphorylated by JNK in response to UV or during thymic selection [23]. Similarly, we recently reported that gliotoxin, a fungal toxin of *Aspergillus fumigatus*, induced apoptosis via JNK1/2-mediated phosphorylation of Bim at three sites (S100, T112 and S114) [24]. Bim phosphorylation by JNK1/2 was shown to increase its binding affinity to Bcl-2-like survival factors which translated into a better activation of Bax/Bak with subsequent cytochrome c release and caspase-3/-7 activation [23], [24]. In agreement with this, we could observe increased caspase-3/-7 levels after stimulation with IL-1β + FasL, and this response was abrogated in Bim^-/-^ hepatocytes as well as in wt hepatocytes treated with the JNK inhibitor SP600125. In addition to Bim, we showed that Bid was crucial for the increased caspase-3/-7 activity. Bid^-/-^ hepatocytes were unable to increase caspase-3/-7 activity upon IL1β + FasL treatment, thus preserving cell viability.

Our experimental observations are supported by a mathematical model, which was established to confirm that our network structure covers the observed effects in all scenarios and explains the underlying mechanism. The model indicates that the sensitizing is due to a depletion of free anti-apoptotic Bcl-2 proteins after tBid and Bim were formed and/or activated in response to IL-1β and FasL treatment. FasL contributes to apoptosis by cleaving full-length Bid into the pro-apoptotic tBid form, whereas IL-1β enhances the pro-apoptotic activity of Bim by JNK1/2-mediated phosphorylation (pBim). tBid and pBim bind to anti-apoptotic Bcl-2 proteins with high affinities. Hence, when cells are treated with FasL or IL-1β alone, either one of the BH3-only proteins is fully sequestered by Bcl-2 proteins. However, if both stimuli are present, the Bcl-2 protein buffer is insufficient to block tBid and pBim simultaneously leading to free amounts of the BH3-only proteins which can then directly activate Bax/Bak and trigger MOMP, cytochrome c release and increased caspase-3 activation. Thus, IL-1β sensitizes hepatocytes to FasL-induced caspase-3/-7 activation by shifting the balance from anti- to pro-apoptotic Bcl-2 proteins. The crucial role of Bid, Bim and JNK1/2 was further emphasized by simulating the knockout and inhibition scenarios. MOMP was ablated in both Bid^-/-^ and Bim^-/-^ knockout cells as well as in wt hepatocytes treated with the JNK inhibitor SP600125. While in Bid^-/-^ cells, blockage of MOMP was because FasL could not trigger tBid formation, in Bim^-/-^ cells and wt hepatocytes treated with SP600125 IL-1β was unable to favour MOMP via Bim phosphorylation. However, the question remained how IL-1β could increase FasL-induced caspase-3/-7 activation but at the same time attenuate FasL-induced cell death.

The threshold for MOMP-activation may significantly vary in individual cells within a population [33]–[35]. The intracellular ratio of pro- and anti-apoptotic Bcl-2 proteins determines the susceptibility of individual cells to apoptotic stimuli [36], [37]. Furthermore, Kallenberger et al. described heterogeneous caspase-8 activation in individual cells following FasL stimulation that critically depended on the quantity of FasL - Fas interaction and DISC formation [29]. We observed that only a fraction of hepatocytes died after 3 h of FasL treatment, whereas others appeared resistant to the stimulus. Our mathematical simulations showed that this heterogeneity may be explained by the dependence of caspase-3 activation and thus cell fate on different initial protein levels of Fas and Bcl-2. Accordingly, IL-1β pre-treatment favors MOMP-mediated apoptosis by influencing the Bcl-2 balance and thus promotes increased caspase-3 activity, but only in those cells which are susceptible to FasL-induced cell death anyway. Thereby, IL-1β pre-incubation leads to higher average caspase-3 levels but not to increased cell death. Our model predicts that pre-incubation with IL-1β changes the ratio of survivors, type I cells and type II cells. This could be verified by monitoring single cell responses to FasL and IL-1β + FasL stimulation distinguishing between type I and type II cells. So far the missing increase of PARP cleavage, the downstream event of caspase-3/-7 activation, supports our hypothesis.

We observed an enhancement of NF-κB DNA binding activity and induction of the NF-κB target gene A20 after treatment with IL-1β + FasL, whereas NF-κB DNA binding activity and A20 mRNA induction was reduced when IL-1β was substituted by TNFα (Figs. 8 and 9A). Enhanced expression of A20 could be confirmed on the protein level (Fig. 9 B-D). This might explain increased cell viability of IL-1β + FasL treated hepatocytes. A20 is an inhibitor of caspase-8 activation and thereby can mediate a protective effect on FasL-induced apoptosis [28]. In our mathematical model, expression of the protective protein X that could be A20 can explain decreased caspase-3 activity in Bid^-/-^ hepatocytes treated with IL-1β + FasL as well as increased cell viability in wt cells after treatment with IL-1β and FasL (Fig. 13B).

In addition to NF-κB activation and increased expression of A20 other survival signaling such as the PI3K/Akt pathway might be crucial for IL-1β-mediated protection. For example, active caspase-3 may trigger Akt activation and protection from apoptosis by proteolytic cleavage of RasGAP to a N-terminal fragment [38]. Furthermore, proteins that directly associate with active caspases and block their proteolytic activity such as the HGF-receptor domain, survivin or XIAP may suppress cell death induced by FasL [12], [39], [40]. Our results with XIAP^-^/^-^ hepatocytes did not reveal any implication of XIAP in the IL-1β–mediated survival response. Whether Akt or other cellular caspase inhibitors are involved in the protective effect remains to be elucidated.

Altogether, we characterized the IL-1β-mediated sensitizing effect on FasL-induced caspase-3/-7 activation via comprehensive studies. Based on our experiments and according to our mathematical model it can be assumed that IL-1β exerts two distinct effects on FasL-induced apoptosis. On one hand it shifts the balance of Bcl-2 proteins to favoring MOMP and increasing the average caspase-3/-7 activity. On the other hand this pro-apoptotic effect is minimized by elevating A20 levels, which may reduce caspase-8 activation and further caspase-3 activation. Therefore, cells that show only a weak response to FasL stimulation and low caspase-8 levels could be rescued by a pre-incubation with IL-1β resulting in elevated cell viability. Moreover, we hypothesize that the seemingly contradictory results of increased caspase-3/-7 activity but decreased cell death do not represent the situation in a single cell but the average of a cell population.

## Supporting Information

S1 Fig **Model simulation of initial IL-1β signaling.** Simulated time courses of the IL-1β receptor complexes, Tab/Tak1 and activated Tak1 (Tak1*).
(TIF)

S2 Fig **Simulated time course of pJNK.**
(TIF)

S3 Fig **Time and concentration dependence of IL-1β pre-treatment on FasL-induced caspase-3/-7 activity.** Primary murine hepatocytes were pre-treated for different times with 20 ng IL-1β (A) or different doses of IL-1β (B) for 12 h and subsequently stimulated with 50 ng/ml FasL for 6 h. Caspase-3/-7 activity was measured by a fluorogenic DEVDase assay (A) Values of three independent experiments ± s.d. are shown. (B) Values of three independent experiments ± s.d. are shown except for treatments with 0.1, 50, and 100 ng/ml, n = 2 (^##^p<0.01, ***p<0.001, IL-1β + FasL versus FasL treated cells).
(TIF)

S4 Fig **Increased FasL-induced PARP cleavage after pre-treatment with TNFα as compared to FasL alone in primary murine hepatocytes.** Whole cell lysates were prepared after pre-treatment of primary murine hepatocytes with TNFα (25 ng/ml) for 12 h or 24 h followed by FasL (50 ng/ml) incubation for further 6 h. PARP cleavage is determined by immunoblotting. Actin is shown as the loading control. A representative immunoblot is presented, n = 3.
(TIF)

S5 Fig **Absence of Bim diminishes the sensitization of FasL-induced apoptosis by TNFα.** Hepatocytes from Bim^-/-^ and wt mice were preteated with TNFα (25 ng/ml) for 12 or 24 h followed by 6 h FasL (50 ng/ml) incubation. Caspase-3/-7 activity (A) and cell viability using the MTT assay (B) were determined. Values are referred to untreated control and represent at least 3 independent experiments ± s.d. (*p<0.01, TNFα + FasL versus FasL treated wt cells).
(TIF)

S6 Fig **Cytochrome c release, caspase-3/-7 activity and DNA fragmentation in hepatocytes treated with TNFα and FasL.** Primary murine hepatocytes were treated with FasL and TNFα + FasL for the indicated times before performing a cytochrome c release assay by ELISA (A), a fluorogenic DEVDase caspase assay (B) or a cell death DNA fragmentation assay (C). Values for the cell death ELISA are referred to the untreated control. Values represent n = 5 for (A), n = 4 for (B) and n = 5 (C) independent experiments ± s.d. (*p<0.05, **p<0.01, ***p<0.001, IL-1β + FasL versus FasL-treated cells at the corresponding time point).
(TIF)

S7 Fig **The sensitizing effect of FasL-induced caspase-3/-7 by IL-1β is independent of XIAP.** Caspase-3/-7 activity was measured in wt and XIAP^-/-^ hepatocytes pre-treated with IL-1β (20 ng/ml) for 12 or 24 h followed by incubation for 6 h with 50 ng/ml FasL (A) and 10 or 25 ng/ml FasL (B). Values represent three independent experiments ± s.d. for (A) and two independent experiments ± s.d. for (B) (*p<0.05, ***p<0.001, IL-1β + FasL versus FasL treatment in wt cells). (C) XIAP protein levels in wt hepatocytes treated with IL-1β, FasL (50 ng/ml) or the combination of both for the indicated times. Actin is shown as loading control. A representative immunoblot is presented, n = 2.
(TIF)

S8 Fig **Structure of the NF-κB module.** The NF-κB module was adopted from Lipniacki et al. [38]. Input of this module is Tak1* that is activated in response to IL-1β and itself activates the IKK complex. The nucleus is modeled as separate compartment with a volume ratio V_Cytosol_∶V_Nucleus_ of 3∶1. Degradation of species is indicated by boxes with dashed borders.
(TIF)

S9 Fig **Simulation of caspase-8 and -3 activities, cytochrome c release and levels of pJNK, Bid, Bim, Bcl-2 and XIAP after treating Bid^-/-^ hepatocytes with IL-1β, FasL or a combination of both.** Simulation results for primary hepatocytes derived from Bid^-/-^ mice after stimulation with IL-1β (A, D), FasL (B, E) and in combination (C, F). IL-1β stimulus is given at time point 0, FasL is added after 12 h. The time course of Bim is invisible, because it overlays with Bcl-2. Other invisible curves are zero. Abbreviations: C8p18, active caspase-8; C3*, active caspase-3; pJNK, phosphorylated JNK.
(TIF)

S10 Fig **Simulation of caspase-8 and -3 activities, cytochrome c release and levels of pJNK, Bid, Bim, Bcl-2 and XIAP after treating Bim^-/-^ hepatocytes with IL-1β, FasL or a combination of both.** Simulation results for primary hepatocytes derived from Bim^-/-^ mice after stimulation with IL-1β (A, D), FasL (B, E) and in combination (C, F). IL-1β stimulus is given at time point 0, FasL is added after 12 h. The time course of Bid is invisible, because it overlays with Bcl-2. Other invisible curves are zero. Abbreviations: C8p18, active caspase-8; C3*, active caspase-3; pJNK, phosphorylated JNK.
(TIF)

S11 Fig **Simulation of caspase-8 and -3 activities, cytochrome c release and levels of pJNK, Bid, Bim, Bcl-2 and XIAP after treating XIAP^-/-^ hepatocytes with IL-1β, FasL or a combination of both.** Simulation results for primary hepatocytes derived from XIAP^-/-^ mice after stimulation with IL-1β (A, D), FasL (B, E) and in combination (C, F). IL-1β stimulus is given at time point 0, FasL is added after 12 h. The time course of Bim is invisible in (D), because it overlays with Bid. Other invisible curves are zero. Abbreviations: C8p18, active caspase-8; C3*, active caspase-3; pJNK, phosphorylated JNK.
(TIF)

S12 Fig **Simulation of caspase-8 and -3 activities, cytochrome c release and levels of pJNK, Bid, Bim, Bcl-2 and XIAP after treating JNK1/2-inhibited wt cells with IL-1β, FasL or a combination of both.** Simulation results for primary hepatocytes treated with the JNK inhibitor SP600125 and with IL-1β (A, D), FasL (B, E) and in combination (C, F). IL-1β stimulus is given at time point 0, FasL is added after 12 h. The time courses of Bid and Bim overlay with Bcl-2 in (D) and the time course of Bid overlays with Bcl-2 in (E) and (F). Other invisible curves are zero. Abbreviations: C8p18, active caspase-8; C3*, active caspase-3; pJNK, phosphorylated JNK.
(TIF)

S13 Fig **Caspase-3 activity is dependent on initial levels of Fas and Bcl-2.** Simulation results for primary hepatocytes upon treatment with FasL (A) and IL-1β + FasL (B) for various initial levels of Fas and anti-apoptotic Bcl-2 proteins (Bcl-2) as representatives for variations at the level of DISC formation and MOMP induction, respectively. Nominal initial conditions are at 100% for both proteins and were varied ±10%.
(TIF)

S1 Table **Species of the IL-1β/FasL model.** Notation of the species of the IL-1β/FasL model. The official symbol and the official full name is taken from the NCBI Gene data base. The according Gene ID is also stated as well as the initial condition (IC). If the model species does not correspond to a gene of the NCBI data base, e.g. in case of modified proteins or complexes, a description is given instead of the official full name.
(PDF)

S2 Table **Parameters of the IL-1β/FasL model.** References for the parameter values listed in this table can be found in S1 Protocol.
(PDF)

S1 Protocol **The file contains all experimental data which is not shown in the manuscript as well as more detailed information on model setup and parameterization.**
(PDF)
