# Supplementary figures and images for: Interleukin-1β Enhances FasL-Induced Caspase-3/-7 Activity without Increasing Apoptosis in Primary Mouse Hepatocytes
Source: PLoS One. 2014 Dec 31;9(12):e115603. doi: 10.1371/journal.pone.0115603 (PMC4281199; doi:10.1371/journal.pone.0115603)

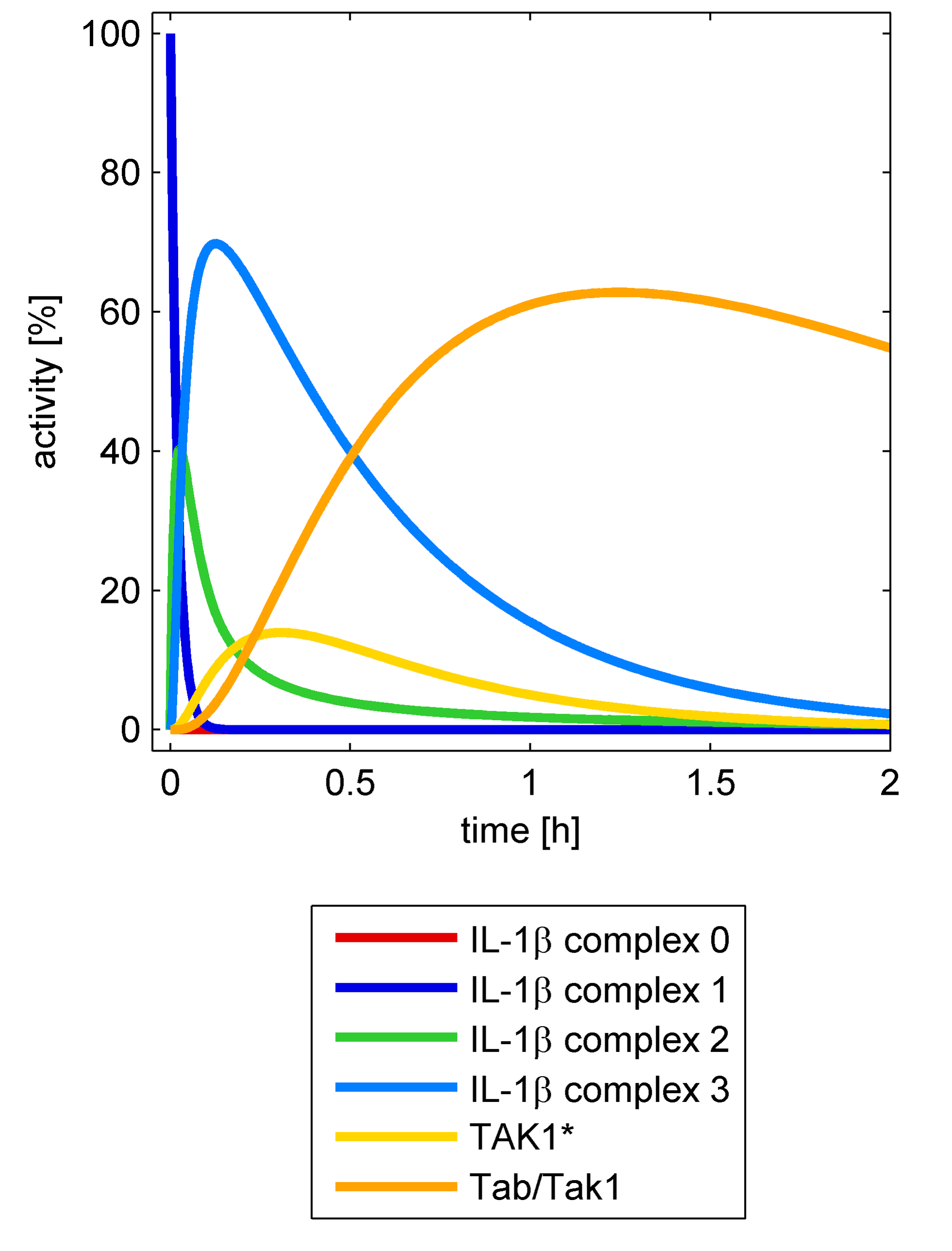

Supplement: S1 Fig — Model simulation of initial IL-1β signaling. Simulated time courses of the IL-1β receptor complexes, Tab/Tak1 and activated Tak1 (Tak1*). (TIF) [file pone.0115603.s001.tif]

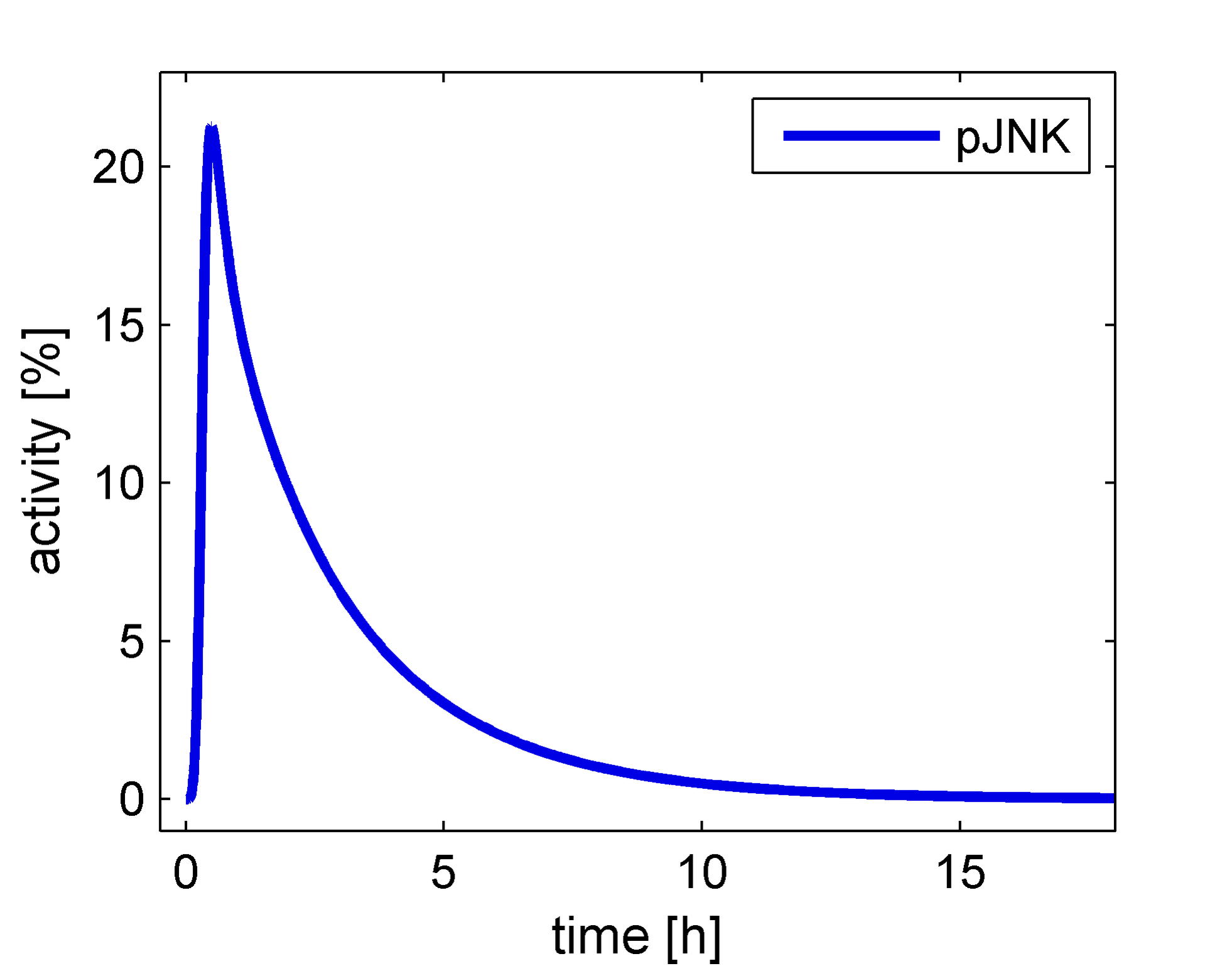

Supplement: S2 Fig — Simulated time course of pJNK. (TIF) [file pone.0115603.s002.tif]

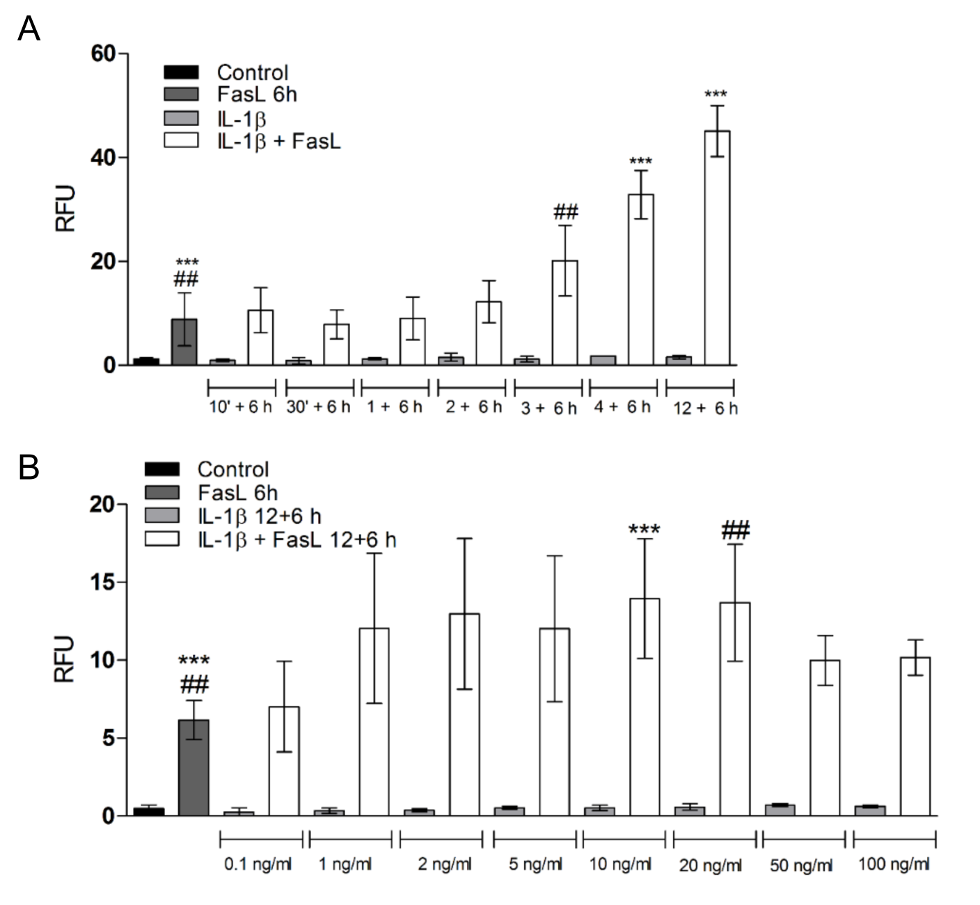

Supplement: S3 Fig — Time and concentration dependence of IL-1β pre-treatment on FasL-induced caspase-3/-7 activity. Primary murine hepatocytes were pre-treated for different times with 20 ng IL-1β (A) or different doses of IL-1β (B) for 12 h and subsequently stimulated with 50 ng/ml FasL for 6 h. Caspase-3/-7 activity was measured by a fluorogenic DEVDase assay (A) Values of three independent experiments ± s.d. are shown. (B) Values of three independent experiments ± s.d. are shown except for treatments with 0.1, 50, and 100 ng/ml, n = 2 (##p<0.01, ***p<0.001, IL-1β + FasL versus FasL treated cells). (TIF) [file pone.0115603.s003.tif]

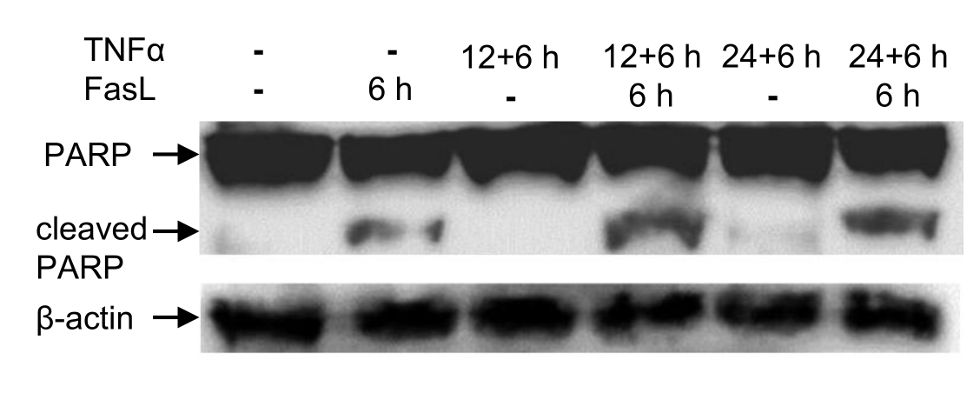

Supplement: S4 Fig — Increased FasL-induced PARP cleavage after pre-treatment with TNFα as compared to FasL alone in primary murine hepatocytes. Whole cell lysates were prepared after pre-treatment of primary murine hepatocytes with TNFα (25 ng/ml) for 12 h or 24 h followed by FasL (50 ng/ml) incubation for further 6 h. PARP cleavage is determined by immunoblotting. Actin is shown as the loading control. A representative immunoblot is presented, n = 3. (TIF) [file pone.0115603.s004.tif]

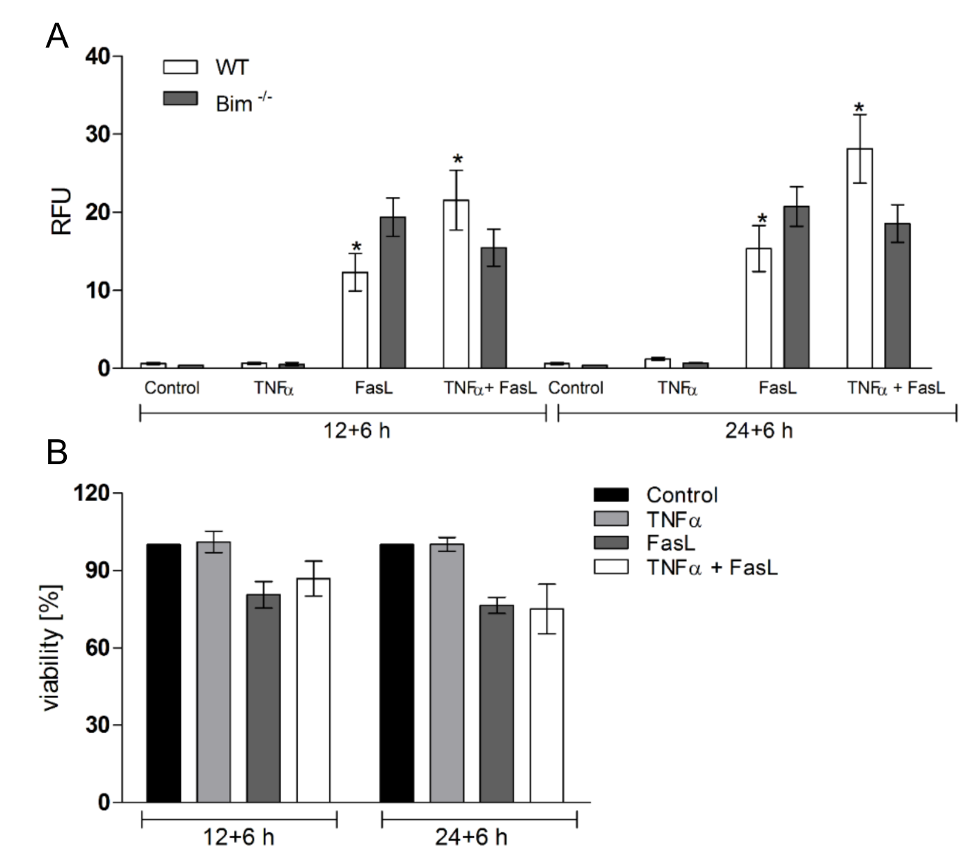

Supplement: S5 Fig — Absence of Bim diminishes the sensitization of FasL-induced apoptosis by TNFα. Hepatocytes from Bim-/- and wt mice were preteated with TNFα (25 ng/ml) for 12 or 24 h followed by 6 h FasL (50 ng/ml) incubation. Caspase-3/-7 activity (A) and cell viability using the MTT assay (B) were determined. Values are referred to untreated control and represent at least 3 independent experiments ± s.d. (*p<0.01, TNFα + FasL versus FasL treated wt cells). (TIF) [file pone.0115603.s005.tif]

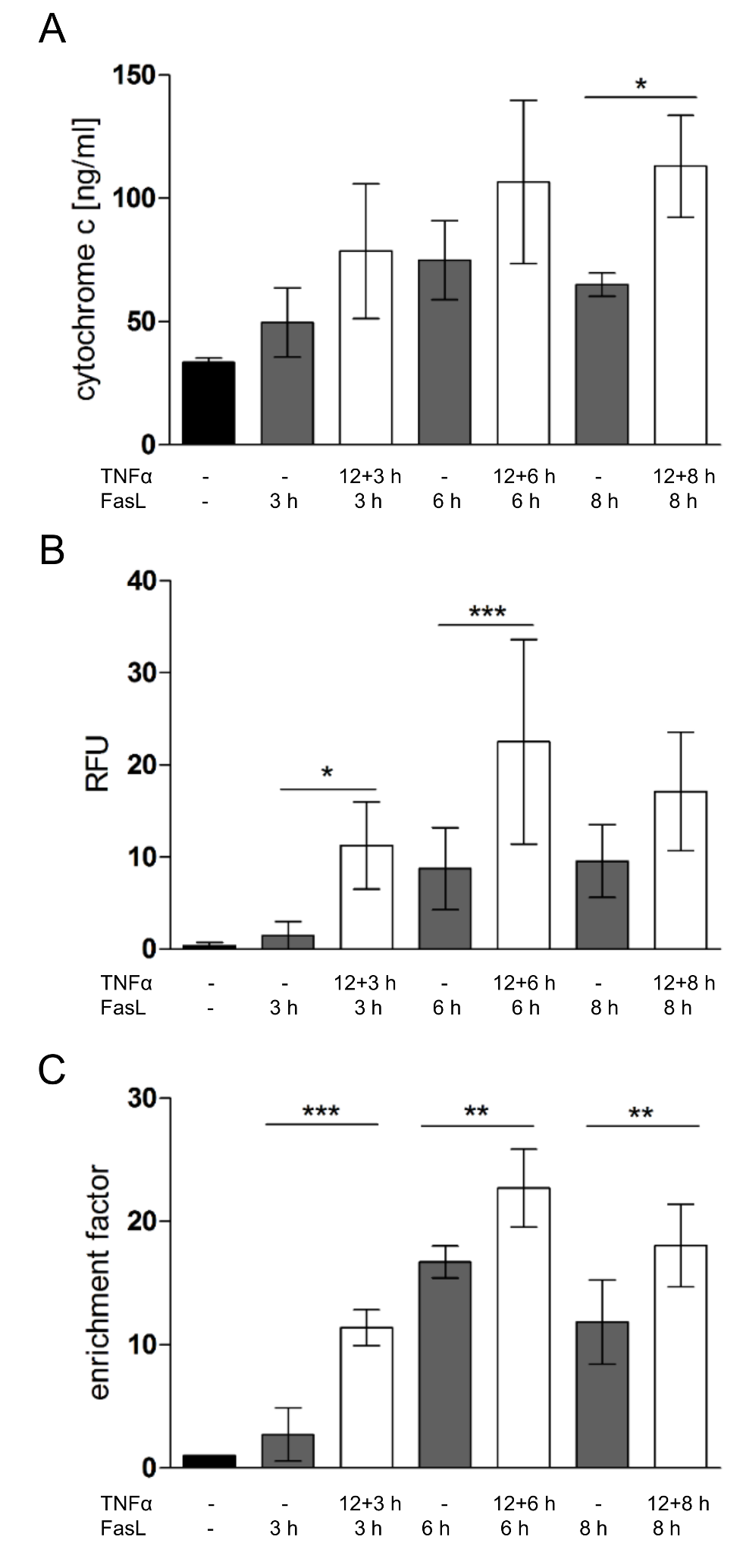

Supplement: S6 Fig — Cytochrome c release, caspase-3/-7 activity and DNA fragmentation in hepatocytes treated with TNFα and FasL. Primary murine hepatocytes were treated with FasL and TNFα + FasL for the indicated times before performing a cytochrome c release assay by ELISA (A), a fluorogenic DEVDase caspase assay (B) or a cell death DNA fragmentation assay (C). Values for the cell death ELISA are referred to the untreated control. Values represent n = 5 for (A), n = 4 for (B) and n = 5 (C) independent experiments ± s.d. (*p<0.05, **p<0.01, ***p<0.001, IL-1β + FasL versus FasL-treated cells at the corresponding time point). (TIF) [file pone.0115603.s006.tif]

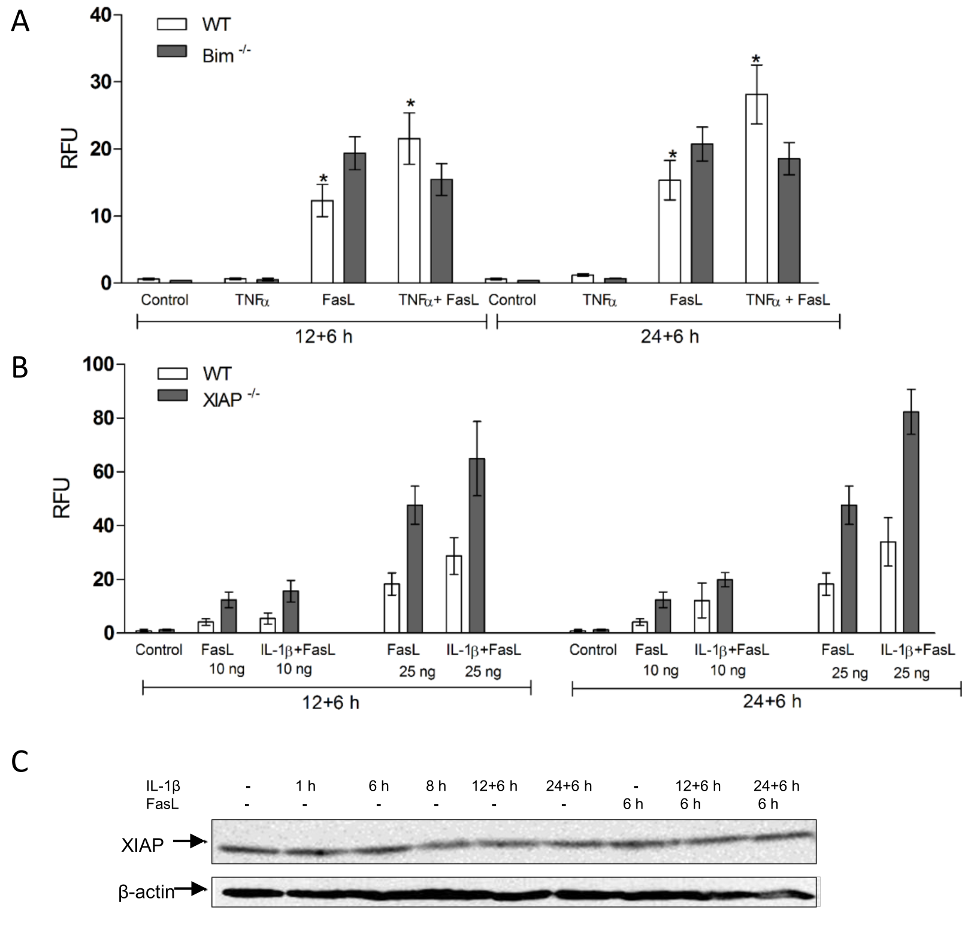

Supplement: S7 Fig — The sensitizing effect of FasL-induced caspase-3/-7 by IL-1β is independent of XIAP. Caspase-3/-7 activity was measured in wt and XIAP-/- hepatocytes pre-treated with IL-1β (20 ng/ml) for 12 or 24 h followed by incubation for 6 h with 50 ng/ml FasL (A) and 10 or 25 ng/ml FasL (B). Values represent three independent experiments ± s.d. for (A) and two independent experiments ± s.d. for (B) (*p<0.05, ***p<0.001, IL-1β + FasL versus FasL treatment in wt cells). (C) XIAP protein levels in wt hepatocytes treated with IL-1β, FasL (50 ng/ml) or the combination of both for the indicated times. Actin is shown as loading control. A representative immunoblot is presented, n = 2. (TIF) [file pone.0115603.s007.tif]

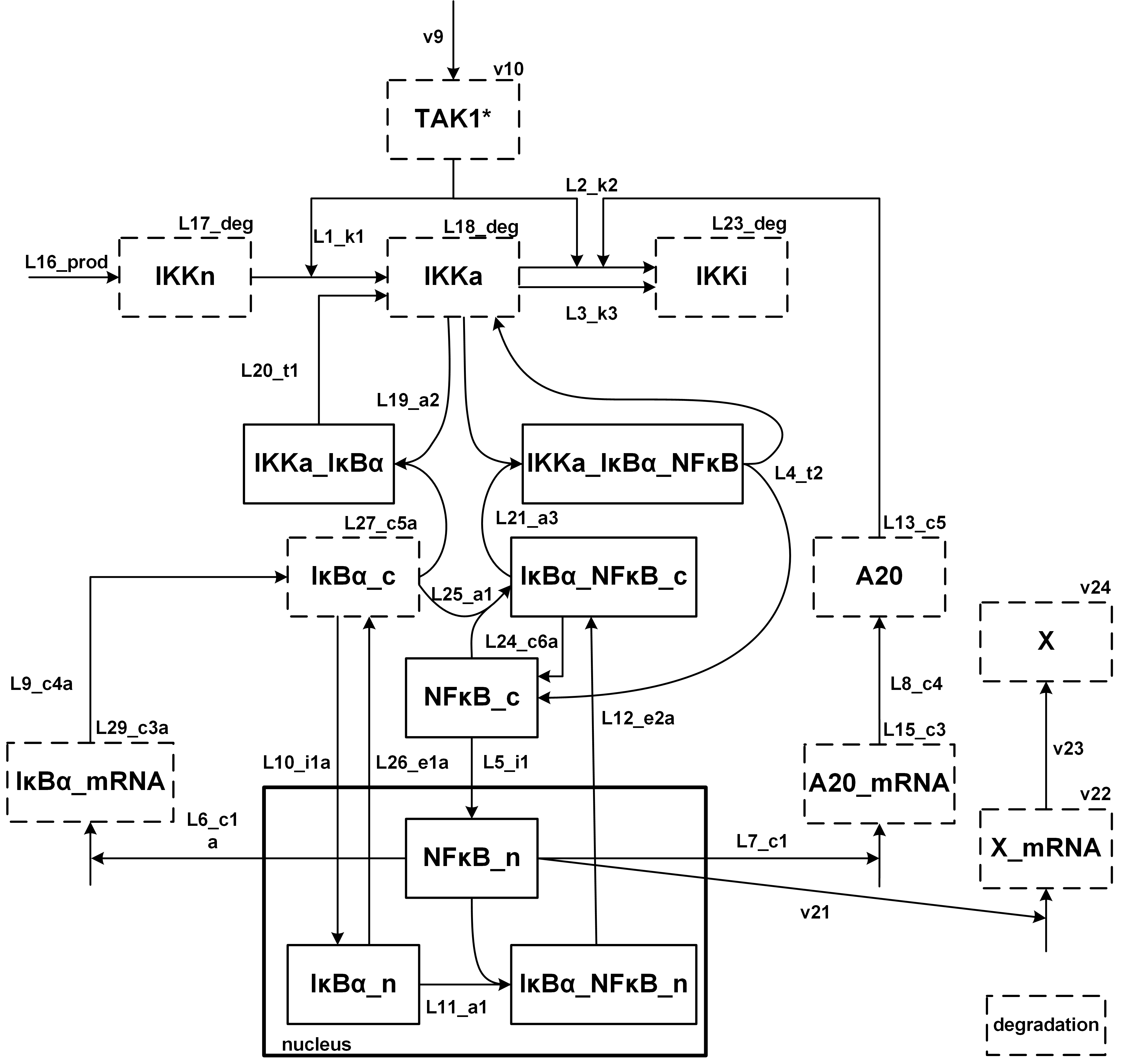

Supplement: S8 Fig — Structure of the NF-κB module. The NF-κB module was adopted from Lipniacki et al. [38]. Input of this module is Tak1* that is activated in response to IL-1β and itself activates the IKK complex. The nucleus is modeled as separate compartment with a volume ratio VCytosol∶VNucleus of 3∶1. Degradation of species is indicated by boxes with dashed borders. (TIF) [file pone.0115603.s008.tif]

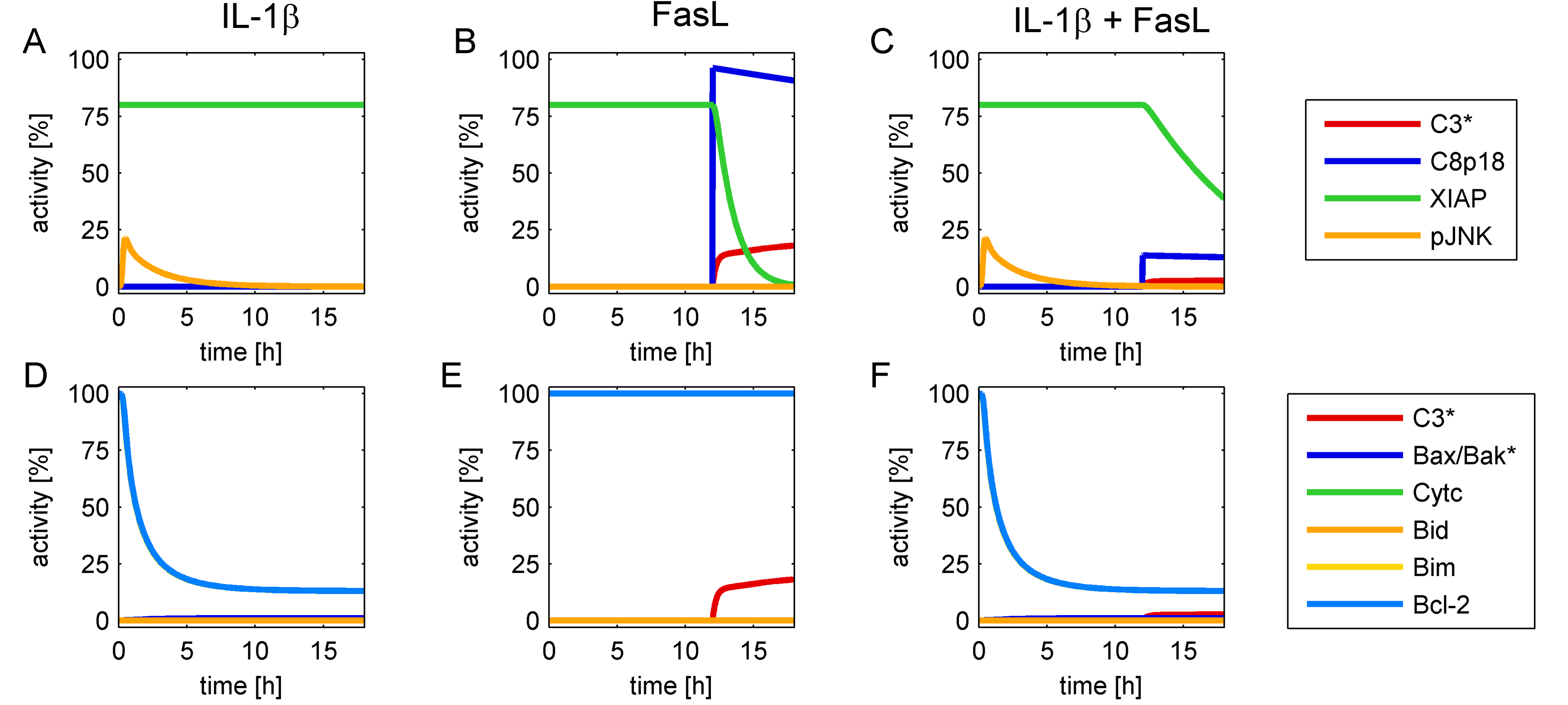

Supplement: S9 Fig — Simulation of caspase-8 and -3 activities, cytochrome c release and levels of pJNK, Bid, Bim, Bcl-2 and XIAP after treating Bid-/- hepatocytes with IL-1β, FasL or a combination of both. Simulation results for primary hepatocytes derived from Bid-/- mice after stimulation with IL-1β (A, D), FasL (B, E) and in combination (C, F). IL-1β stimulus is given at time point 0, FasL is added after 12 h. The time course of Bim is invisible, because it overlays with Bcl-2. Other invisible curves are zero. Abbreviations: C8p18, active caspase-8; C3*, active caspase-3; pJNK, phosphorylated JNK. (TIF) [file pone.0115603.s009.tif]

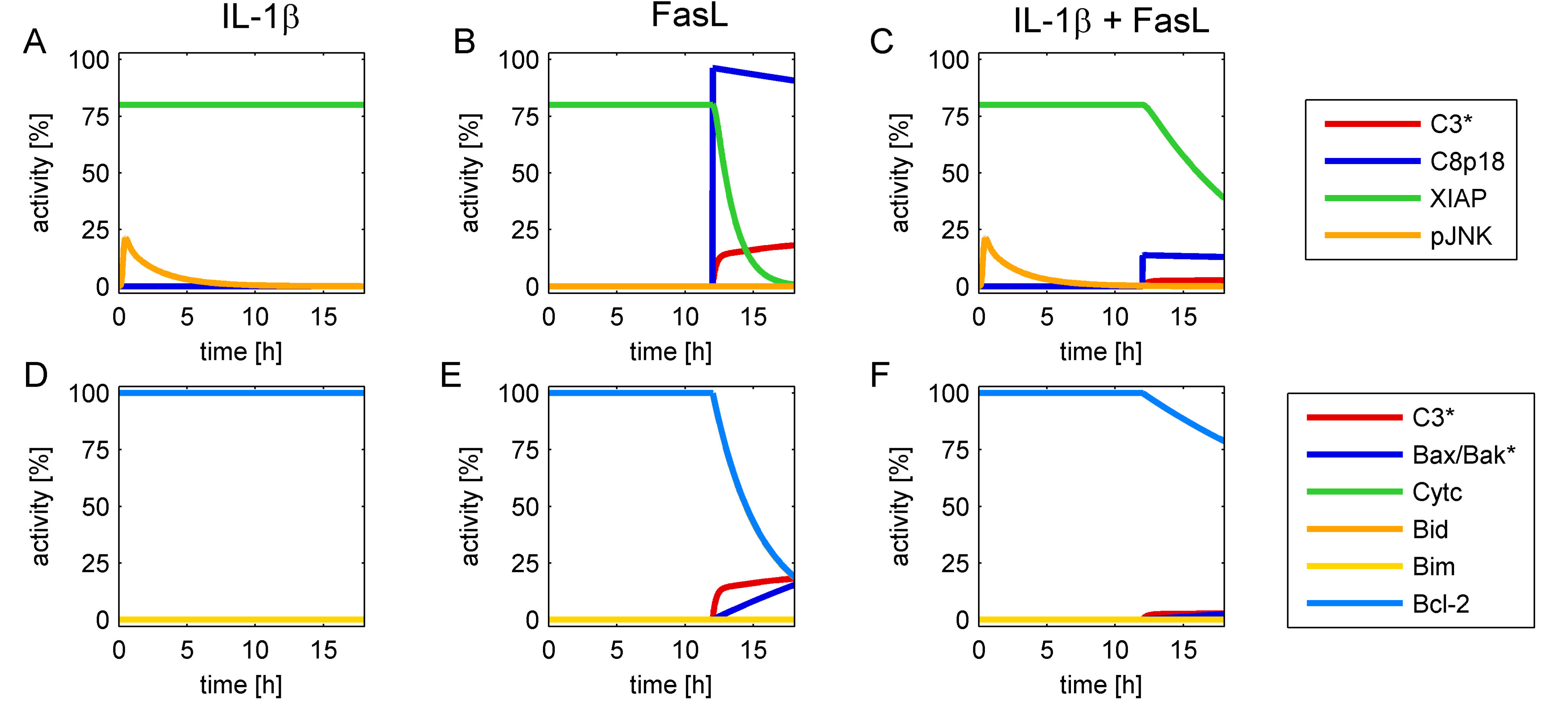

Supplement: S10 Fig — Simulation of caspase-8 and -3 activities, cytochrome c release and levels of pJNK, Bid, Bim, Bcl-2 and XIAP after treating Bim-/- hepatocytes with IL-1β, FasL or a combination of both. Simulation results for primary hepatocytes derived from Bim-/- mice after stimulation with IL-1β (A, D), FasL (B, E) and in combination (C, F). IL-1β stimulus is given at time point 0, FasL is added after 12 h. The time course of Bid is invisible, because it overlays with Bcl-2. Other invisible curves are zero. Abbreviations: C8p18, active caspase-8; C3*, active caspase-3; pJNK, phosphorylated JNK. (TIF) [file pone.0115603.s010.tif]

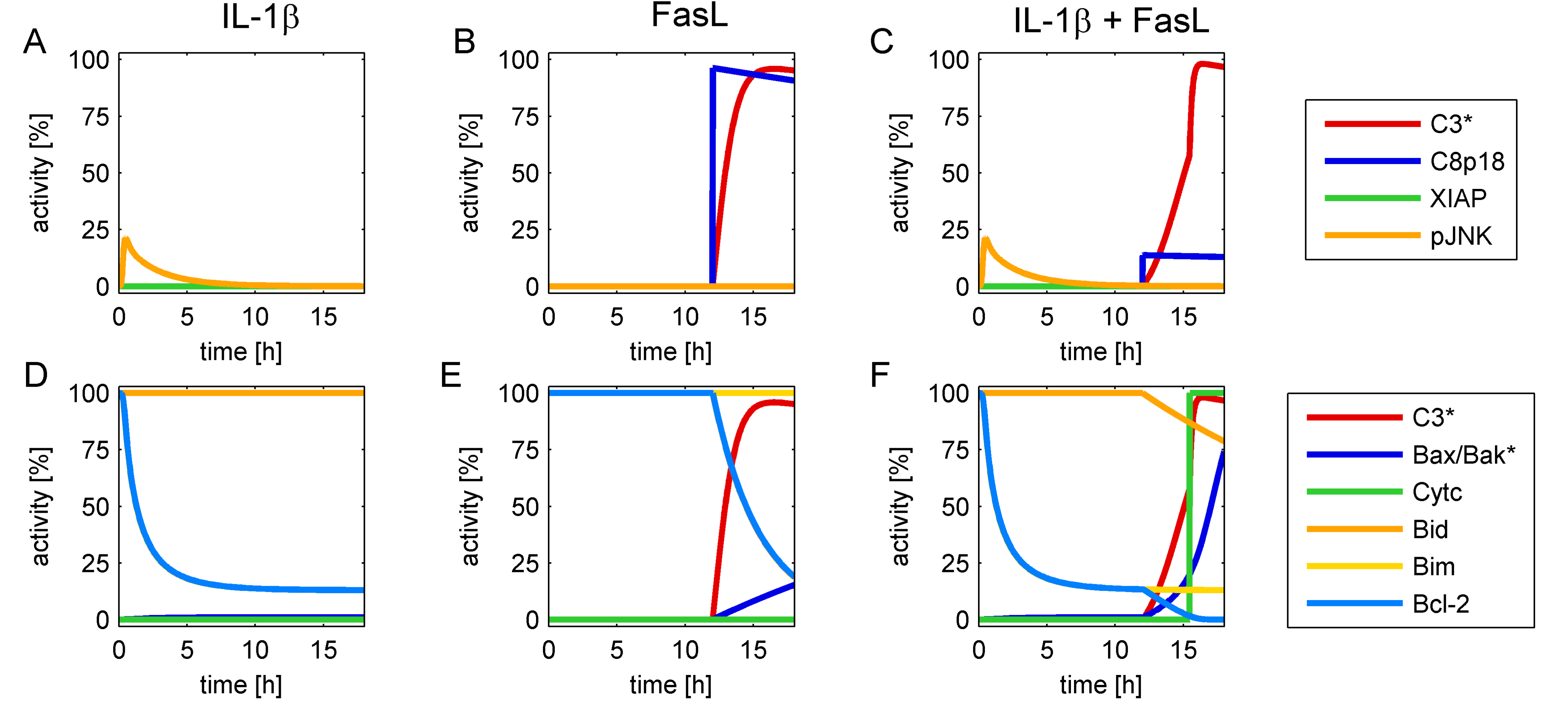

Supplement: S11 Fig — Simulation of caspase-8 and -3 activities, cytochrome c release and levels of pJNK, Bid, Bim, Bcl-2 and XIAP after treating XIAP-/- hepatocytes with IL-1β, FasL or a combination of both. Simulation results for primary hepatocytes derived from XIAP-/- mice after stimulation with IL-1β (A, D), FasL (B, E) and in combination (C, F). IL-1β stimulus is given at time point 0, FasL is added after 12 h. The time course of Bim is invisible in (D), because it overlays with Bid. Other invisible curves are zero. Abbreviations: C8p18, active caspase-8; C3*, active caspase-3; pJNK, phosphorylated JNK. (TIF) [file pone.0115603.s011.tif]

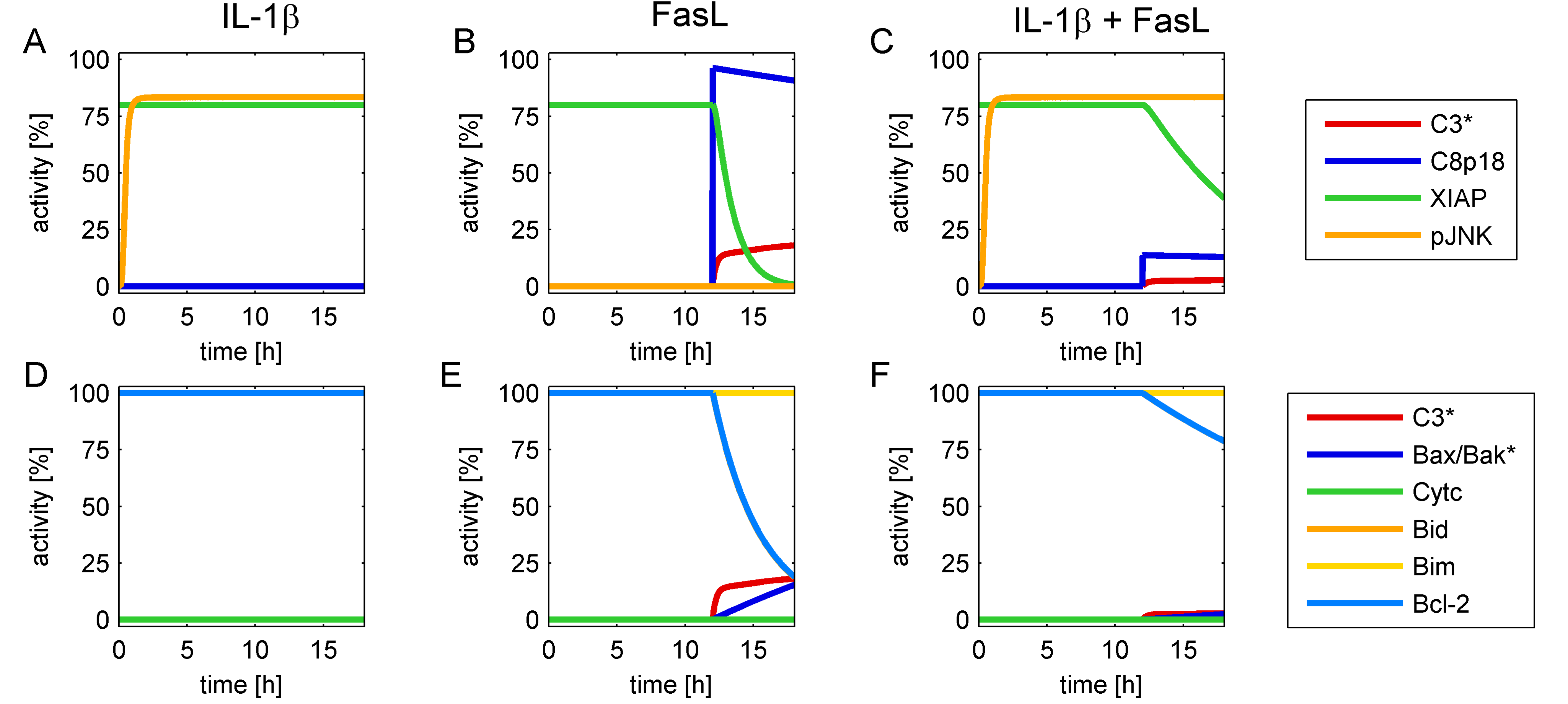

Supplement: S12 Fig — Simulation of caspase-8 and -3 activities, cytochrome c release and levels of pJNK, Bid, Bim, Bcl-2 and XIAP after treating JNK1/2-inhibited wt cells with IL-1β, FasL or a combination of both. Simulation results for primary hepatocytes treated with the JNK inhibitor SP600125 and with IL-1β (A, D), FasL (B, E) and in combination (C, F). IL-1β stimulus is given at time point 0, FasL is added after 12 h. The time courses of Bid and Bim overlay with Bcl-2 in (D) and the time course of Bid overlays with Bcl-2 in (E) and (F). Other invisible curves are zero. Abbreviations: C8p18, active caspase-8; C3*, active caspase-3; pJNK, phosphorylated JNK. (TIF) [file pone.0115603.s012.tif]

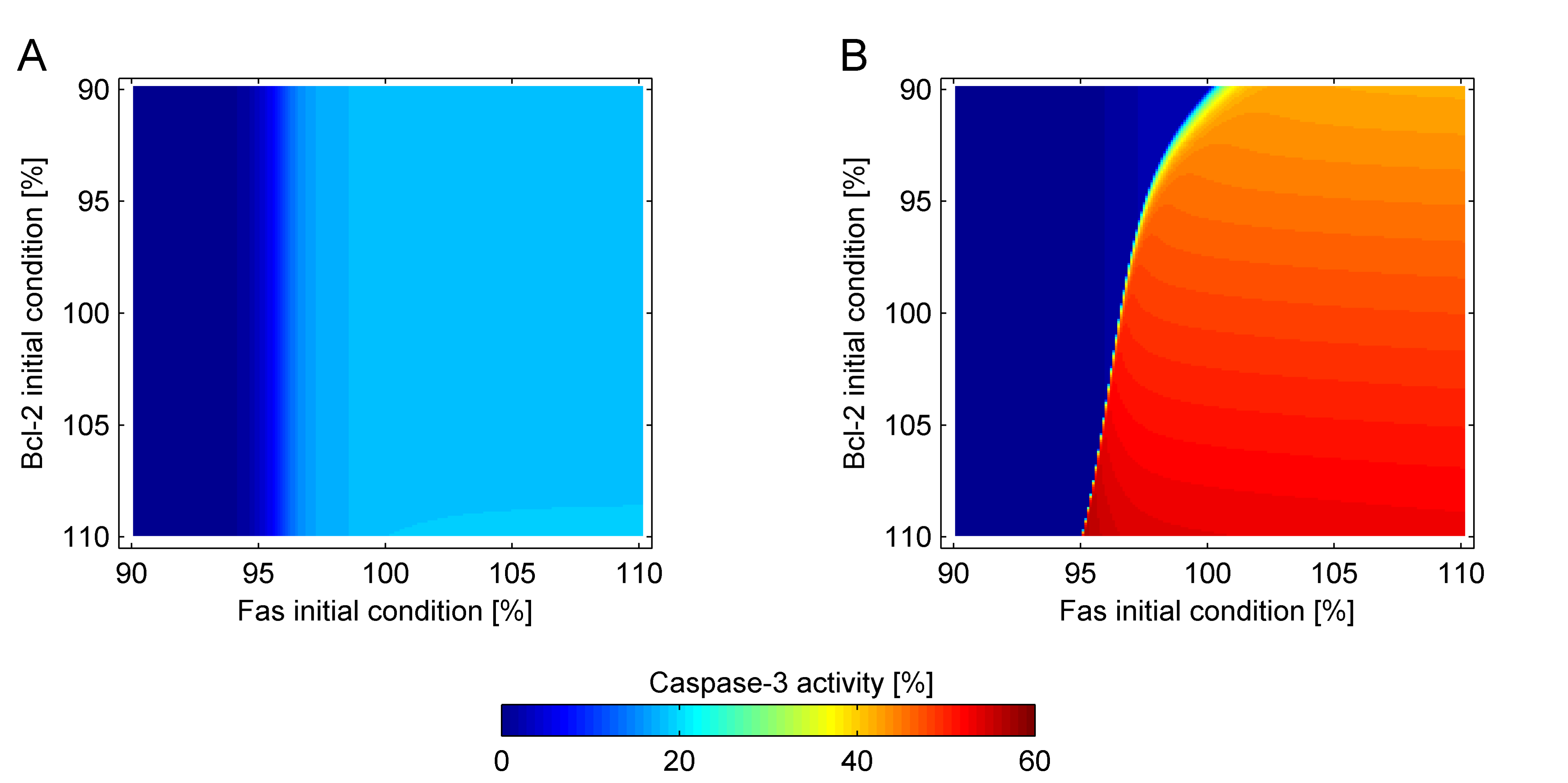

Supplement: S13 Fig — Caspase-3 activity is dependent on initial levels of Fas and Bcl-2. Simulation results for primary hepatocytes upon treatment with FasL (A) and IL-1β + FasL (B) for various initial levels of Fas and anti-apoptotic Bcl-2 proteins (Bcl-2) as representatives for variations at the level of DISC formation and MOMP induction, respectively. Nominal initial conditions are at 100% for both proteins and were varied ±10%. (TIF) [file pone.0115603.s013.tif]
