# Supplementary material for: Interleukin-1β Enhances FasL-Induced Caspase-3/-7 Activity without Increasing Apoptosis in Primary Mouse Hepatocytes
Source: PLoS One. 2014 Dec 31;9(12):e115603. doi: 10.1371/journal.pone.0115603 (PMC4281199; doi:10.1371/journal.pone.0115603)
Supplement: S1 Table — Species of the IL-1β/FasL model. Notation of the species of the IL-1β/FasL model. The official symbol and the official full name is taken from the NCBI Gene data base. The according Gene ID is also stated as well as the initial condition (IC). If the model species does not correspond to a gene of the NCBI data base, e.g. in case of modified proteins or complexes, a description is given instead of the official full name. (PDF) [file pone.0115603.s014.pdf]

Table S1: Species of the IL-1β/FasL model. Notation of the species of the IL-1β/FasL model. The official symbol and the official full name is taken from the NCBI Gene data base. The according Gene ID is also stated as well as the initial condition (IC). If the model species does not correspond to a gene of the NCBI data base, e.g. in case of modified proteins or complexes, a description is given instead of the official full name.

species	official symbol	official full name / description	NCBI Gene ID	IC [%]
A20	Tnfaip3	tumor necrosis factor, alpha-induced protein 3	21929	4.59003 * 10^{-03}
A20_mRNA	-	mRNA encoding A20	-	0
Bax/Bak	Bax, Bak1	Bax (BCL2-associated X protein) and Bak (BCL2-antagonist/killer 1)	12028, 12018	100
Bax/Bak*	-	activated Bax/Bak oligomers	-	0
Bcl-2	Bcl2	B cell leukemia/lymphoma 2	12043	100
Bid	Bid	BH3 interacting domain death agonist	12122	100
Bim	Bcl2l11	BCL2-like 11 (apoptosis facilitator)	12125	100
C3	Casp3	caspase 3 (procaspase)	12367	100
C3*	-	activated caspase 3	-	0
C8p18	-	fully activated caspase 8 (after second cleavage step)	-	0
C8p43	-	caspase 8 (after first cleavage step)	-	0
C8p55	Casp8	caspase 8 (procaspase)	12370	100
cFLIP	Cflar	CASP8 and FADD-like apoptosis regulator	12633	100
Cytc free	Cycs	cytochrome c, somatic (released into the cytosol)	13063	0
DcFLIP	-	inactive complex of DISC and cFLIP	-	0
DISC	-	death-inducing signaling complex comprising FasR/CD95, FADD, and caspase 8	-	0
DISCtrans-Act	-	DISC trans activity - dummy species determining the threshold of DISC molecules that is necessary for the final caspase-8 cleavage step into the p18 fragment according to Kallenberger et al., 2014	-	0
FADD	Fadd	Fas (TNFRSF6)-associated via death domain	14082	100
FasL	Fasl	Fas ligand (TNF superfamily, member 6)	14103	0
Fas	Fas	Fas (TNF receptor superfamily member 6)	14102	100

species	official symbol	official full name / description	NCBI Gene ID	IC [%]
IκBα_c	Nfkbia	nuclear factor of kappa light polypeptide gene enhancer in B cells inhibitor, alpha (cytosolic)	18035	1.99001 $* 10^{-03}$
IκBα_mRNA	-	mRNA encoding IκBα	-	0
IκBα_n	Nfkbia	nuclear factor of kappa light polypeptide gene enhancer in B cells inhibitor, alpha (nuclear)	18035	2.29475 $* 10^{-03}$
IκBα _NFκB_c	-	cytosolic complex of NFκB and IκBα	-	5.89005 $* 10^{-02}$
IκBα _NFκB_n	-	nuclear complex of NFκB and IκBα	-	8.42637 $* 10^{-05}$
IKKa	Chuk, Ikbkb, Ikbkg	activated form of the IKK complex comprising IKKα/Chuk (conserved helix-loop-helix ubiquitous kinase), IKKβ/Ikbkb (inhibitor of kappaB kinase beta) and IKKγ/Ikbkg (inhibitor of kappaB kinase gamma)	12675, 16150, 16151	0
IKKa_IκBα	-	inactive complex comprising the IKK complex and IκBα	-	0
IKKa_IκBα _NFκB	-	inactive complex comprising the IKK complex, IκBα and NFκB	-	0
IKKi	-	inhibited form of the IKK complex	-	0
IKKn	-	neutral form of the IKK complex	-	0.2
IL-1β	Il1b	interleukin 1 beta	16176	0/1
IL-1β complex 0	Il1r1, Il1rap, Myd88, Tollip, Irak1, Irak4, Traf6	precursors of the IL-1β complex 1: IL-1R/Il1r1 (interleukin 1 receptor, type I), IL-1RAcP/Il1rap (interleukin 1 receptor accessory protein), MyD88/Myd88 (myeloid differentiation primary response gene 88), Tollip (toll interacting protein), Irak1/4 (interleukin-1 receptor-associated kinase 1/4), Traf6 (TNF receptor-associated factor 6)	16177, 16180, 17874, 54473, 16179, 266632, 22034	100
IL-1β complex 1	-	IL-1β complex 1 comprising IL-1β, IL-1R1, IL-1RAcP, MyD88, Tollip, Irak1, Irak4, and Traf6	-	0
IL-1β complex 2	-	IL-1β complex 2 comprising Irak1, Traf6, Tab1/2, and Tak1	-	0
IL-1β complex 3	-	IL-1β complex 3 comprising Traf6, Tab1/2, and Tak1	-	0
inFasL	-	dummy species indicating if cells are stimulated with FasL or not	-	0/1
JNK	Mapk8/9	mitogen-activated protein kinase 8/9	26419, 26420	100
MKK7	Map2k7	mitogen-activated protein kinase kinase 7	26400	100

species	official symbol	official full name / description	NCBI Gene ID	IC [%]
NFκB_c	Rela	v-rel reticuloendotheliosis viral oncogene homolog A (avian) (cytosolic)	19697	$3.37055 * 10^{-04}$
NFκB_n	Rela	v-rel reticuloendotheliosis viral oncogene homolog A (avian) (nuclear)	19697	$2.20322 * 10^{-03}$
pBim	-	phosphorylated, active form of Bim	-	0
phosphatase	-	a MAP kinase phosphatase (activated)	-	0
pJNK	-	phosphorylated, active form of JNK	-	0
pMKK7	-	phosphorylated, active form of MKK7	-	0
prophosphatase	-	a MAP kinase phosphatase	-	100
Smac/ DIABLO	Diablo	diablo homolog (Drosophila)	66593	0
Smac-XIAP	-	inactive complex comprising Smac/DIABLO and XIAP	-	0
Tab/Tak1	Tab1/2, Map3k7	complex comprising Tab1/2 (TGF-beta activated kinase 1/2 / MAP3K7 binding protein 1/2) and Tak1/Map3k7 (mitogen-activated protein kinase kinase kinase 7)	66513, 68652, 26409	100
Tak1*	Map3k7	activated form of Tak1/Map3k7 (mitogen-activated protein kinase kinase kinase 7)	26409	0
tBid	-	truncated, active form of Bid	-	0
X	-	unknown protein X	-	0
X_mRNA	-	mRNA encoding protein X	-	0
XIAP	Xiap	X-linked inhibitor of apopotsis	11798	80
