# Supplementary material for: Interleukin-1β Enhances FasL-Induced Caspase-3/-7 Activity without Increasing Apoptosis in Primary Mouse Hepatocytes
Source: PLoS One. 2014 Dec 31;9(12):e115603. doi: 10.1371/journal.pone.0115603 (PMC4281199; doi:10.1371/journal.pone.0115603)
Supplement: S2 Table — Parameters of the IL-1β/FasL model. References for the parameter values listed in this table can be found in S1 Protocol. (PDF) [file pone.0115603.s015.pdf]

Table S2: Parameters of the IL-1β/FasL model. References for the parameter values listed in this table can be found in Supporting Information (Protocol S1).

parameter	value	unit	description	reference
k_{v1}	0.001	$AU^{-1}\ h^{-1}$	constitutive production of IL-1β complex 0	[1, 14]
k_{v2}	0.001	h^{-1}	degradation of IL-1β complex 0	[1, 14]
k_{v3}	50	$AU^{-1}\ h^{-1}$	IL-1β-induced assembly of IL-1β complex 1	[14]
k_{v4}	0.05	h^{-1}	degradation of IL-1β complex 1	[1, 14]
k_{v5}	0.5	$AU^{-1}\ h^{-1}$	assembly of IL-1β complex 2	[14]
k_{v6}	0.05	h^{-1}	degradation of IL-1β complex 2	[1, 14]
k_{v7}	2	h^{-1}	assembly of IL-1β complex 3	[14]
k_{v8}	0.05	h^{-1}	degradation of IL-1β complex 3	[1, 14]
k_{v9}	8	h^{-1}	activation of Tak1	[14]
k_{v10}	0.4	h^{-1}	degradation of Tak1*	[14]
k_{v11}	0.05	$AU^{-1}\ h^{-1}$	activation of MKK7 by Tak1*	[1]
k_{v12}	0.2	$AU^{-1}\ h^{-1}$	activation of JNK by pMKK7	Fig. 4A
k_{v13}	4	h^{-1}	inactivation of pJNK	Fig. 4A
k_{v14}	0.02	$AU^{-1}h^{-1}$	inhibition of XIAP by released Smac/DIABLO	-
$k1_{v15}$	0.8	AU^{-1}	first caspase-8 cleavage step into the p43 fragment within DISC	[1]
$k2_{v15}$	0.25	$AU\ h^{-1}$	inhibition of caspase-8 cleavage by A20	-
k_{v16}	2	$AU^{-2}\ h^{-2}$	second caspase-8 cleavage step into the fully active p18 fragment requiring DISC trans activity	-
k_{v17}	0.01	h^{-1}	degradation of C8p43	[1]
k_{v18}	0.01	h^{-1}	degradation of C8p18	[1]
k_{v19}	0.003	$AU^{-1}\ h^{-1}$	cleavage of Bid into the active fragment tBid by C8p18	-
k_{v20}	0.006	$AU^{-1}\ h^{-1}$	activation of caspase-3 by C8p18	-
k_{v21}	0.0018	h^{-1}	NF-κB-induced synthesis of protein X mRNA	[4]
k_{v22}	1.44	h^{-1}	degradation of protein X mRNA	[4]
k_{v23}	1800	h^{-1}	synthesis of protein X	[4]
k_{v24}	1.08	h^{-1}	degradation of protein X	[4]

parameter	value	unit	description	reference
k_{Sv3}	0.04	AU^{-1} h^{-1}	phosphorylation of Bim by pJNK	[1]
k_{Sv4}	0.001	h^{-1}	degradation of pBim	[1]
k_{Sv5}	1	AU^{-1} h^{-1}	inhibition of pBIM via binding of Bcl-2	[1]
k_{Sv6}	0.005	AU^{-1} h^{-1}	formation of Bax/Bak* complexes induced by pBim	[1]
k_{Sv10}	0.001	h^{-1}	degradation of tBid	[1]
k_{Sv11}	1	AU^{-1} h^{-1}	inhibition of tBid via binding of Bcl-2	[1]
k_{Sv12}	0.1	AU^{-1} h^{-1}	formation of Bax/Bak* complexes induced by tBid	[1]
k_{Sv13}	0.0001	h^{-1}	inactivation of Bax/Bak*	[1]
k_{Sv15}	0.05	AU^{-1} h^{-1}	activation of caspase-3 within the apoptosome mediated by cytochrome c release	[1]
k_{Sv16}	0.007	AU^{-1} h^{-1}	autoactivation of caspase-3	[1]
k_{Sv17}	0.01	h^{-1}	degradation of active caspase-3*	[1]
k_{Sv18}	0.05	AU^{-1} h^{-1}	inhibition of caspase-3* by XIAP	[1]
k_{Sv25}	0.05	AU^{-1} h^{-1}	activation of the MAP kinase phosphatase by pJNK	[1]
k_{Sv26}	0.05	h^{-1}	inactivation of the MAP kinase phosphatase	[1]
k_{Sv27}	2	AU^{-1} h^{-1}	dephosphorylation of pMKK7 by the MAP kinase phosphatase	[1]
k_{Sv40}	8	AU^{-1} h^{-1}	inhibition of caspase-8 activation at the DISC by cFLIP	[1]
k_{Sv42}	0.008	h^{-1}	decomposition of DcFLIP into DISC and cFLIP	[1]
k_{Sv46}	0.05	h^{-1}	degradation of DISC	[1]
k_{Sv47}	0.005	AU^{-2} h^{-2}	FasL-induced assembly of the DISC	[1]
k_{L1_k1}	9	AU^{-1} h^{-1}	activation of IKK by Tak1*	[4]
k_{L2_k2}	360	AU^{-2} h^{-2}	inhibition of IKK by Tak1* and A20	[4]
k_{L3_k3}	5.4	h^{-1}	spontaneous inhibition of IKK	[4]
k_{L4_t2}	360	h^{-1}	liberation of IKK and NF-κB	[4]
k_{L5_i1}	9	h^{-1}	translocation of cytosolic NF-κB to the nucleus	[4]
k_{L6_c1a}	0.0018	h^{-1}	NF-κB-induced synthesis of IκBα mRNA	[4]

parameter	value	unit	description	reference
k_{L7_c1}	0.0018	h^{-1}	NF-κB-induced synthesis of A20 mRNA	[4]
k_{L8_c4}	1800	h^{-1}	synthesis of A20	[4]
k_{L9_c4a}	1800	h^{-1}	synthesis of IκBα	[4]
k_{L10_i1a}	3.6	h^{-1}	translocation of cytosolic IκBα to the nucleus	[4]
k_{L11_a1}	1800	AU^{-1} h^{-1}	inhibition of nuclear NF-κB via binding of IκBα	[4]
k_{L12_e2a}	36	h^{-1}	translocation of nuclear IκBα_NF-κB to the cytosol	[4]
k_{L13_c5}	1.08	h^{-1}	degradation of A20	[4]
k_{L15_c3}	1.44	h^{-1}	degradation of A20 mRNA	[4]
k_{L16_prod}	0.09	AU^{-1} h^{-1}	constitutive production of IKKn	[4]
k_{L17_deg}	0.45	h^{-1}	degradation of IKKn	[4]
k_{L18_deg}	0.45	h^{-1}	degradation of IKKa	[4]
k_{L19_a2}	720	AU^{-1} h^{-1}	complex formation of IκBα and IKKa	[4]
k_{L20_t1}	360	h^{-1}	liberation of IKKa	[4]
k_{L21_a3}	3600	AU^{-1} h^{-1}	binding of IKKa to the complex of IκBα and NF-κB	[4]
k_{L23_deg}	0.45	h^{-1}	degradation of IKKi	[4]
k_{L24_c6a}	0.072	h^{-1}	liberation of NF-κB	[4]
k_{L25_a1}	1800	AU^{-1} h^{-1}	inhibition of cytosolic NF-κB via binding of IκBα	[4]
k_{L26_e1a}	1.8	h^{-1}	translocation of nuclear IκBα to the cytosol	[4]
k_{L27_c5a}	0.36	h^{-1}	degradation of IκBα	[4]
k_{L29_c3a}	1.44	h^{-1}	degradation of IκBα mRNA	[4]
