# Supplementary material for: Interleukin-1β Enhances FasL-Induced Caspase-3/-7 Activity without Increasing Apoptosis in Primary Mouse Hepatocytes
Source: PLoS One. 2014 Dec 31;9(12):e115603. doi: 10.1371/journal.pone.0115603 (PMC4281199; doi:10.1371/journal.pone.0115603)
Supplement: S1 Protocol — The file contains all experimental data which is not shown in the manuscript as well as more detailed information on model setup and parameterization. (PDF) [file pone.0115603.s016.pdf]

Supporting Information

"Interleukin-1β enhances FasL-induced caspase-3/-7 activity without increasing apoptosis in primary mouse hepatocytes"

Anna Lutz[1*], Julia Sanwald[2*], Maria Thomas[3], Ronny Feuer[2], Oliver Sawodny[2], Michael Ederer[2], Christoph Borner[4,5,6], Matjaz Humar[1], Irmgard Merfort[1,5]

[1] Department of Pharmaceutical Biology and Biotechnology, Albert Ludwigs University Freiburg, Stefan-Meier-Str. 19, 79104 Freiburg, Germany

[2] Institute for System Dynamics, University of Stuttgart, 70550 Stuttgart, Germany

[3] Dr. Margarete Fischer-Bosch Institute of Clinical Pharmacology, Auerbachstr. 112, 70376 Stuttgart, Germany

[4] Institute of Molecular Medicine and Cell Research, Albert Ludwigs University Freiburg, Stefan-Meierstr. 17, 79104 Freiburg, Germany

[5] Spemann Graduate School of Biology and Medicine (SGBM), Albert Ludwigs University Freiburg, Albertstr. 19a, 79106 Freiburg, Germany

[6] Bioss – Centre for Biological Signaling Studies, Albert Ludwigs University Freiburg, Albertstr. 19, 79106 Freiburg, Germany

Experimental Methods

DEVDase assay

The activity of the executioner caspase-3/-7 was measured by the fluorogenic DEVDase assay as previously described [1]. Primary mouse hepatocytes (1 x 10^6) were treated with cytokines (IL-1β, TNFα) and/or in combination with FasL (generated by Neuro-2 A cells) for the indicated times. After the incubation the cells were detached, washed with PBS and centrifuged (2150 x g at 4 °C for 3 min). The pellet was homogenized in 50 μl lysis buffer (50 mM Hepes-KOH, 2.5 mM MgCl2, 2.5 mM EGTA, 12 mM DTT supplemented with the protease inhibitors 12 μg/ml aprotinin, 12 μg/ml leupeptin, 0.5 μg/ml pepstatin, 0.125 μM PMSF, 1.5 μg/ml cytochalasin B) by freeze-thaw cycles and finally centrifuged at 20'800 g at 4 °C for 10 min. The caspase-3/-7 activity assay was performed using the DEVD-AMC (Alexis) substrate at a concentration of 200 nM. 8 μl of the protein extract were mixed with 90 μl assay buffer (50 mM Hepes-KOH, 12 mM DTT) and the DEVD-AMC substrate. Subsequently the fluorescence was measured for 40 cycles and the slope was calculated. The relative fluorescence unit (RFU) was determined by the ratio of the slope and the protein concentration of each sample, measured by Bradford assay (Biorad). To compare different experiments, RFU sample values were referred to negative control (untreated cells).

Viability assay (MTT assay)

The MTT assay was carried out according to the method described by Mosmann, 1983 [2]. Briefly, primary murine hepatocytes were differently treated and subsequently incubated with a 0.1 mg/ml 3-(4,5-dimethylthiazol-2-yl)-2,5-diphenyl tetrazolium bromide (MTT) - PBS (Roth) solution in William's E Medium for 2 h at 37 °C. The excess of MTT was removed and the formazan crystals formed were dissolved in DMSO. The

samples were transferred in a 96-well plate; diluted 1:5 in PBS and the absorbance of purple formazan, proportional to the number of viable cells, was measured at 595 nm with a microplate reader. The absorption was referred to untreated control, respectively.

Cell Death detection ELISA

Hepatocytes (1 x 10^6) were treated with the stimuli at different times. Subsequently, the cells were washed with PBS, detached and incubated in 500 µl lysis buffer for 30 min at RT. The lysate was centrifuged at 2150 x g for 10 min and the supernatant was used to prepare a 1:20 dilution in lysis buffer. Samples and controls were transferred to the streptavidin-coated microplate and incubated with the immunoreagent (containing incubation buffer, anti-histone-biotin and anti-DNA-POD) for 2 h at 25 °C and shaking at 300 rpm. Afterwards, the wells were washed and incubated with ATBS-solution for 20 min gently shaken at 300 rpm. The reaction was stopped by adding ATBS-stop-solution and the absorbance was measured at 405 nm and 490 nm as reference wavelength. The sample values were referred to untreated control.

Electrophoretic mobility shift assay (EMSA)

Nuclear extracts were prepared as described by Schreiber et al., 1989 [3]. For EMSAs, equal amounts of nuclear proteins (5 µg) were filled up to 12 µL with H_2O and a reaction mixture containing of Poly (dIdC) (1 µg/µl, Roche), BSA (10 µg/µl), Buffer D (20 mM HEPES pH 7.9, 20 % glycerol, 100 mMKCl, 0.5 mM EDTA, 0.25 % NP-40, 2 mM DTT, 0,1 % PMSF), Buffer F (20 % Ficoll 400, 100 mM HEPES pH 7.9, 300 mMKCl, 10 mM DTT, 0.1 % PMSF) and 25 ng of a [γ33P]-labeled NF-κB binding oligonucleotide were added and incubated for 30 min at RT. Subsequently, the extracts were separated using a non-denaturating 6% polyacrylamide gel, the gel was dryed on a vacuum gel dryer for 60 min at 80 °C and exposed to Imaging Plate (BAS-MS 2340, Fujifilm) overnight which

was finally analyzed using a FLA-3000 (Fujifilm).

Modeling

Model Development

The model presented here is partially based on the TNFα/FasL model by Schlatter et al. [1]. The FasL signaling module was adopted from there and complemented with a module describing IL-1β signaling as well as with a NF-κB module from Lipniacki et al. [4] that was already part of the TNFα/FasL model [1]. All reactions from [4] are adopted without any modifications and are labeled L_i. Three modifications were made in the FasL signaling module: (1) Smac/DIABLO was introduced as pro-apoptotic factor that is released from the mitochondria in a similar manner as cytochrome c [5, 6, 7] and impedes the activity of XIAP (v14) [8, 9, 10]. (2) An IL-1β-induced protective effect was observed in our experiments that is mediated by a yet unknown mechanism. Therefore, a protein X was added to the model that is induced by NF-κB following IL-1β stimulation and inhibits caspase-8 activation at DISC (v15). A hypothesis is that this protein X is A20, since A20 is known to protect cells from Fas-mediated apoptosis via inhibition of caspase-8 activation [11]. Also, our results demonstrate that A20 is significantly stronger induced in response to IL-1β than after TNFα stimulation (Fig. 9). So far, this is only a hypothesis and needs further testing and thus we refer to this protein as protein X. Nevertheless, parameters for synthesis and degradation of protein X and its mRNA (k_{v21}, k_{v22}, k_{v23}, k_{v24}) are taken from synthesis and degradation of A20 and A20 mRNA (k_{L7_c1}, k_{L15_c3}, k_{L8_c4}, k_{L13_c5}). (3) Caspase-8 activation was modeled in more detail according to the mechanism proposed by Kallenberger et al. [12]. The first caspase-8 cleavage step into the p43 fragment occurs by an intramolecular process within

DISC (v15). This activation can be impeded by protein X. The second caspase-8 cleavage step into the fully active p18 fragment occurs in an interdimeric manner and, therefore, requires interaction of several DISC molecules. This mechanism was implemented in a simplified manner by realizing a threshold concentration of DISC that is necessary for the second caspase-8 cleavage step. For this purpose a dummy species (DISCtransAct) was introduced that represents DISC trans activity. This species is activated, if enough DISCs are formed and promotes v16. Due to this modification, the reactions v8, v9, v14 and v22 of the TNFα/FasL model also needed to be adapted and are now labeled v17, v19, v20 and v15, respectively. The parameter value for the degradation of activated caspase-8 (C8*) of the TNFα/FasL model (k_8) is now used for both the degradation of the p43 as well as for the p18 fragment of caspase-8 (new parameters k_{v18} and k_{v17}). All other reactions of the TNFα/FasL model were adopted without modification and are labeled Sv_i. All modified or new reactions are label v_i.

The overall model comprises 53 species, 69 reactions, and 70 parameters. It is based on ordinary differential equations (ODEs) and mass action kinetics and was implemented using Copasi [13]. The structure of the resulting model is given in Fig. 10. The integrated NF-κB module is summarized as single gray box for clarity; a more detailed representation is displayed in Fig. S8. All ODEs are given in the following Section and the species with their initial conditions (ICs) are listed in Table S1. In general, ICs of precursors and proteins in the inactive state are set to 100 %, whereas ICs of the activated form of proteins are set to 0 % according to [1]. The ICs of all species that were already part of the TNFα/FasL model were retained with one expectation, namely FADD. The IC of FADD in the TNFα/FasL model was set to 200 % as it served as a common pool for TNFα complex 2 and DISC assembly without competitive behavior [1]. Since FADD only represents a precursor for formation of DISC now in the IL-1β/FasL model, the IC was set to 100 % according to all other precursor species. Parameterization is explained

on page 12 and following. For the reactions labeled Sv_i and L_i that were adopted without any modification also the parameter values were retained. A list of all parameters of the IL-1β/FasL model is given in Table S2.

IL-1β and FasL constitute inputs of the overall model activating their respective signaling pathways that converge on the level of Bax/Bak activation. The inputs represent variables having constant values of either 0 or 1 representing the different stimulation of cells with one or both of these factors. Stimulation with FasL is modeled using an approximated step function resulting in the transition from 0 % FasL to 100 % FasL at 12 h after start of simulation according to the experimental setup if the input variable 'inFasL' is set to 1. Activated caspase-3 (C3*) is regarded as model output. Cytosol and nucleus are modeled as separate compartments assuming the volume ratio $V_{Cytosol}$:$V_{Nucleus}$ to be 3:1 for murine hepatocytes according to [1].

Since receptor recruitment occurs quite fast [14] compared to the overall observed time scale of the model, the individual dynamics of complex formation can be neglected and the involved species can be pooled for simplification. Thus, the IL-1β receptor IL-1R1, the adapter molecules IL-1RAcP, MyD88 and Tollip as well as IRAK4, IRAK1 and TRAF6 were grouped together into 'IL-1β complex 0' that is constitutively produced (v1) and degraded (v2) over time to maintain a basal level in unstimulated cells. Binding of IL-1β induces transition to the IL-1β complex 1 (v3). IL-1β complex 2 is assembled via interaction with Tab and Tak1 (v5) which are already present as a complex at the plasma membrane (Tab/Tak1). Subsequently, the IL-1β complex 3 is formed (v7) leading to release of active Tak1 (Tak1*) into the cytosol (v9). The receptor complexes as well as Tak1* are constantly degraded over time (v4, v6, v8, v10). Tak1* activates MKK7 (v11) which then phosphorylates JNK (v12). Phosphorylated JNK (pJNK) phosphorylates Bim (Sv3) and mobilizes a phosphatase (Sv25) that in turn deactivates pMKK7 (Sv27). This phosphatase is not further specified, but regulation

of MAP kinase activity by protein phosphatases is accepted as common scheme [15]. Candidates for JNK phosphatases are both serine/threonine and tyrosine phosphatases as well as dual specificity phosphatases such as MKP-1 [16]. In addition, active phosphatase and pJNK recover over time to inactive prophosphatase (Sv26) and JNK (v13), respectively. Furthermore, activated Tak1* was shown to activate NF-κB in response to IL-1β via activation of IKK [17]. A detailed description of the reactions of the NF-κB module can be found in [4].

FasL stimulation induces assembly of Fas and FADD into DISC (Sv47). DISC formation induces the first caspase-8 cleavage step (v15) if not inhibited by cFLIP (Sv40). This activation can be impeded by protein X that is induced in response to IL-1β stimulation and NF-κB activation (v23) and constantly degraded (v24). The complex DcFLIP can recover to free cFLIP and DISC (Sv42) and DISC is continuously degraded (Sv46). The second caspase-8 cleavage step (v16) results in fully activated C8p18 that is released into the cytosol and activates both caspase-3 (v20) and Bid (Sv9). C8p43 and C8p18 are continuously degraded (v17, v18). Caspase-3 activation can also occur via released cytochrome c (Sv15) or via autoactivation (Sv16). Activated caspase-3 (C3*) can be inhibited by XIAP (Sv18) and is degraded over time (Sv17).

As already mentioned, the signaling pathways induced by IL-1β and FasL converge at the level of Bax/Bak activation and mitochondrial outer membrane perforation (MOMP). Both pBim and tBid induced by IL-1β and FasL stimulation, respectively, act on Bax/Bak activation and oligomerization (Sv6, Sv12) if not sequestered by anti-apoptotic Bcl-2 family members such as Bcl-2, Bcl-xL or Mcl-1 (Sv5, Sv11). Since the anti-apoptotic Bcl-2 proteins all exhibit similar function, i.e. inhibition of pro-apoptotic family members [18], only one representative anti-apoptotic Bcl-2 protein is modeled. pBim and tBid are both continuously degraded (Sv4, Sv10) and activated Bax/Bak* can recover over time to inactive Bax/Bak (Sv13). Once enough pores are formed within

the mitochondrial membrane, cytochrome c release occurs in a rapid, complete and kinetically invariant manner [19]. Additionally, Smac/DIABLO is released from the mitochondria in a similar time course [6, 5] that subsequently binds to and inhibits XIAP (v14) [8, 9]. In the model, the release of apoptogenic factors from mitochondria was realized using an approximated step function leading to release of 100 % cytochrome c and Smac/DIABLO into the cytosol when a critical threshold of 20 % BaxBak activation is reached. This is in accordance to the extended TNFα/FasL model [1] and was lowered compared to the earlier model [20] because studies suggest that only weak increase in translocation and activation of Bax is sufficient to cause MOMP [21].

Model Equations

$$\frac{d}{dt}\, c_{\text{A20}}(t) = k_{L8\_c4} \cdot c_{\text{A20\_mRNA}}(t) - k_{L13\_c5} \cdot c_{\text{A20}}(t) \tag{1}$$

$$\frac{d}{dt}\, c_{\text{A20\_mRNA}}(t) = k_{L7\_c1} \cdot c_{\text{NF}\kappa\text{B\_n}}(t) - k_{L15\_c3} \cdot c_{\text{A20\_mRNA}}(t) \tag{2}$$

$$\begin{aligned}\frac{d}{dt}\, c_{\text{Bax/Bak}}(t) = {} & k_{Sv13} \cdot c_{\text{Bax/Bak*}}(t) - k_{Sv12} \cdot c_{\text{Bax/Bak}}(t) \cdot c_{\text{tBid}}(t) \\ & - k_{Sv6} \cdot c_{\text{Bax/Bak}}(t) \cdot c_{\text{pBim}}(t)\end{aligned} \tag{3}$$

$$\begin{aligned}\frac{d}{dt}\, c_{\text{Bax/Bak*}}(t) = {} & k_{Sv12} \cdot c_{\text{Bax/Bak}}(t) \cdot c_{\text{tBid}}(t) + k_{Sv6} \cdot c_{\text{Bax/Bak}}(t) \cdot c_{\text{pBim}}(t) \\ & - k_{Sv13} \cdot c_{\text{Bax/Bak*}}(t)\end{aligned} \tag{4}$$

$$\frac{d}{dt}\, c_{\text{Bcl-2}}(t) = -k_{Sv5} \cdot c_{\text{pBim}}(t) \cdot c_{\text{Bcl-2}}(t) - k_{Sv11} c_{\text{tBid}}(t) \cdot c_{\text{Bcl-2}}(t) \tag{5}$$

$$\frac{d}{dt}\, c_{\text{Bid}}(t) = -k_{v19} \cdot c_{\text{Bid}}(t) \cdot c_{\text{C8p18}}(t) \tag{6}$$

$$\frac{d}{dt}\, c_{\text{Bim}}(t) = -k_{Sv3} \cdot c_{\text{Bim}}(t) \cdot c_{\text{pJNK}}(t) \tag{7}$$

$$\begin{aligned}\frac{d}{dt}\, c_{\text{C3}}(t) = {} & -k_{Sv15} \cdot c_{\text{C3}}(t) \cdot c_{\text{Cytc,free}}(t) - k_{Sv16} \cdot c_{\text{C3}}(t) \cdot c_{\text{C3*}}(t) \\ & - k_{v20} \cdot c_{\text{C3}}(t) \cdot c_{\text{C8p18}}(t)\end{aligned} \tag{8}$$

$$
\begin{aligned}
\frac{d}{dt}\, c_{\mathrm{C3^*}}(t) = {} & k_{Sv15} \cdot c_{\mathrm{C3}}(t) \cdot c_{\mathrm{Cytc,free}}(t) + k_{Sv16} \cdot c_{\mathrm{C3}}(t) \cdot c_{\mathrm{C3^*}}(t) \\
& + k_{v20} \cdot c_{\mathrm{C3}}(t) \cdot c_{\mathrm{C8p18}}(t) - k_{Sv17} \cdot c_{\mathrm{C3^*}}(t) \\
& - k_{Sv18} \cdot c_{\mathrm{C3^*}}(t) \cdot c_{\mathrm{XIAP}}(t)
\end{aligned} \tag{9}
$$

$$
\frac{d}{dt}\, c_{\mathrm{C8p18}}(t) = k_{v16} \cdot c_{\mathrm{C8p43}}(t) \cdot c_{\mathrm{DISC}}(t) \cdot c_{\mathrm{DISCtransAct}}(t) - k_{v18} \cdot c_{\mathrm{C8p18}}(t) \tag{10}
$$

$$
\begin{aligned}
\frac{d}{dt}\, c_{\mathrm{C8p43}}(t) = {} & k1_{v15} \cdot c_{\mathrm{C8p55}}(t) \cdot c_{\mathrm{DISC}}(t) \cdot \frac{k2_{v15}}{c_{\mathrm{A20}}(t)} - k_{v17} \cdot c_{\mathrm{C8p43}} \\
& - k_{v16} \cdot c_{\mathrm{C8p43}}(t) \cdot c_{\mathrm{DISC}}(t) \cdot c_{\mathrm{DISCtransAct}}(t)
\end{aligned} \tag{11}
$$

$$
\frac{d}{dt}\, c_{\mathrm{C8p55}}(t) = k1_{v15} \cdot c_{\mathrm{C8p55}}(t) \cdot c_{\mathrm{DISC}}(t) \cdot \frac{k2_{v15}}{c_{\mathrm{A20}}(t)} \tag{12}
$$

$$
\frac{d}{dt}\, c_{\mathrm{cFLIP}}(t) = k_{Sv42} \cdot c_{\mathrm{DcFLIP}}(t) - k_{Sv40} \cdot c_{\mathrm{DISC}}(t) \cdot c_{\mathrm{cFLIP}}(t) \tag{13}
$$

$$
c_{\mathrm{Cytc,free}}(t) = (1 + \tanh(30 * (c_{\mathrm{Bax/Bak^*}}(t) - 20))) * 50 \tag{14}
$$

$$
\frac{d}{dt}\, c_{\mathrm{DcFLIP}}(t) = k_{Sv40} \cdot c_{\mathrm{DISC}}(t) \cdot c_{\mathrm{cFLIP}}(t) - k_{Sv42} \cdot c_{\mathrm{DcFLIP}}(t) \tag{15}
$$

$$
\begin{aligned}
\frac{d}{dt}\, c_{\mathrm{DISC}}(t) = {} & k_{Sv42} \cdot c_{\mathrm{DcFLIP}}(t) + k_{Sv47} \cdot c_{\mathrm{Fas}}(t) \cdot c_{\mathrm{FasL}}(t) \cdot c_{\mathrm{FADD}}(t) \\
& - k_{Sv40} \cdot c_{\mathrm{DISC}}(t) \cdot c_{\mathrm{cFLIP}}(t) - k_{Sv46} \cdot c_{\mathrm{DISC}}(t)
\end{aligned} \tag{16}
$$

$$
c_{\mathrm{DISCtransAct}}(t) = (1 + \tanh(30 * (c_{\mathrm{DISC}}(t) - 2))) * 20 \tag{17}
$$

$$
\frac{d}{dt}\, c_{\mathrm{FADD}}(t) = -k_{Sv47} \cdot c_{\mathrm{Fas}}(t) \cdot c_{\mathrm{FasL}}(t) \cdot c_{\mathrm{FADD}}(t) \tag{18}
$$

$$
c_{\mathrm{FasL}}(t) = \frac{1}{2} * (1 + \tanh(30 * (t - 12))) \tag{19}
$$

$$
\frac{d}{dt}\, c_{\mathrm{Fas}}(t) = -k_{Sv47} \cdot c_{\mathrm{Fas}}(t) \cdot c_{\mathrm{FasL}}(t) \cdot c_{\mathrm{FADD}}(t) \tag{20}
$$

$$
\begin{aligned}
\frac{d}{dt}\, c_{\mathrm{I\kappa B\alpha\_c}}(t) = {} & k_{L9\_c4a} \cdot c_{\mathrm{I\kappa B\alpha\_mRNA}}(t) + k_{L26\_e1a} \cdot c_{\mathrm{I\kappa B\alpha\_n}}(t) \\
& - k_{L10\_i1a} \cdot c_{\mathrm{I\kappa B\alpha\_c}}(t) - k_{L19\_a2} \cdot c_{\mathrm{I\kappa B\alpha\_c}}(t) \cdot c_{\mathrm{IKKa}}(t) \\
& - k_{L25\_a1} \cdot c_{\mathrm{I\kappa B\alpha\_c}}(t) \cdot c_{\mathrm{NF\kappa B\_c}}(t) - k_{L27\_c5a} \cdot c_{\mathrm{I\kappa B\alpha\_c}}(t)
\end{aligned} \tag{21}
$$

$$
\frac{d}{dt}\, c_{\mathrm{I\kappa B\alpha\_mRNA}}(t) = k_{L6\_c1a} \cdot c_{\mathrm{NF\kappa B\_n}}(t) - k_{L29\_c3a} \cdot c_{\mathrm{I\kappa B\alpha\_mRNA}}(t) \tag{22}
$$

$$
\begin{aligned}
\frac{d}{dt}\, c_{\mathrm{I\kappa B\alpha\_n}}(t) = {} & k_{L10\_i1a} \cdot c_{\mathrm{I\kappa B\alpha\_c}}(t) - k_{L26\_e1a} \cdot c_{\mathrm{I\kappa B\alpha\_n}}(t) \\
& - k_{L11\_a1} \cdot V_{\mathrm{Cyt:Nuc}} \cdot c_{\mathrm{I\kappa B\alpha\_n}}(t) \cdot c_{\mathrm{NF\kappa B\_n}}(t)
\end{aligned} \tag{23}
$$

$$\frac{d}{dt}\, c_{\text{IκBα\_NFκB\_c}}(t) = k_{L12\_e2a} \cdot c_{\text{IκBα\_NFκB\_n}}(t) + k_{L25\_a1} \cdot c_{\text{NFκB\_c}}(t) \cdot c_{\text{IκBα\_c}}(t) - k_{L21\_a3} \cdot c_{\text{IKKa}}(t) \cdot c_{\text{ IκBα\_NFκB\_c}}(t) - k_{L24\_c6a} \cdot c_{\text{ IκBα\_NFκB\_c}}(t) \tag{24}$$

$$\frac{d}{dt}\, c_{\text{IκBα\_NFκB\_n}}(t) = k_{L11\_a1} \cdot V_{\text{Cyt:Nuc}} \cdot c_{\text{IκBα\_n}}(t) \cdot c_{\text{NFκB\_n}}(t) - k_{L12\_e2a} \cdot c_{\text{IκBα\_NFκB\_n}}(t) \tag{25}$$

$$\frac{d}{dt}\, c_{\text{IKKa}}(t) = k_{L1\_k1} \cdot c_{\text{IKKn}}(t) \cdot c_{\text{Tak1*}}(t) + k_{L4\_t2} \cdot c_{\text{IKKa\_IκBα\_NFκB}}(t) + k_{L20\_t1} \cdot c_{\text{IKKa\_IκBα}}(t) - k_{L2\_k2} \cdot c_{\text{IKKa}}(t) \cdot c_{\text{Tak1*}}(t) \cdot c_{\text{A20}}(t) - k_{L3\_k3} \cdot c_{\text{IKKa}}(t) - k_{L18\_deg} \cdot c_{\text{IKKa}}(t) - k_{L19\_a2} \cdot c_{\text{IKKa}}(t) \cdot c_{\text{IκBα\_c}}(t) - k_{L21\_a3} \cdot c_{\text{IKKa}}(t) \cdot c_{\text{ IκBα\_NFκB\_c}}(t) \tag{26}$$

$$\frac{d}{dt}\, c_{\text{IKKa\_IκBα}}(t) = k_{L19\_a2} \cdot c_{\text{IKKa}}(t) \cdot c_{\text{IκBα\_c}}(t) - k_{L20\_t1} \cdot c_{\text{IKKa\_IκBα}}(t) \tag{27}$$

$$\frac{d}{dt}\, c_{\text{IKKa\_IκBα\_NFκB}}(t) = k_{L21\_a3} \cdot c_{\text{IKKa}}(t) \cdot c_{\text{ IκBα\_NFκB\_c}}(t) - k_{L4\_t2} \cdot c_{\text{IKKa\_IκBα\_NFκB}}(t) \tag{28}$$

$$\frac{d}{dt}\, c_{\text{IKKi}}(t) = k_{L2\_k2} \cdot c_{\text{IKKa}}(t) \cdot c_{\text{Tak1*}}(t) \cdot c_{\text{A20}}(t) + k_{L3\_k3} \cdot c_{\text{IKKa}}(t) - k_{L23\_deg} \cdot c_{\text{IKKi}}(t) \tag{29}$$

$$\frac{d}{dt}\, c_{\text{IKKn}}(t) = k_{L16\_prod} - k_{L1\_k1} \cdot c_{\text{IKKn}}(t) \cdot c_{\text{Tak1*}}(t) - k_{L17\_deg} \cdot c_{\text{IKKn}}(t) \tag{30}$$

$$\frac{d}{dt}\, c_{\text{IL-1βcomplex0}}(t) = k_{v1} - k_{v2} \cdot c_{\text{IL-1βcomplex0}}(t) - k_{v3} \cdot c_{\text{IL-1βcomplex0}}(t) \cdot c_{\text{IL-1β}}(t) \tag{31}$$

$$\frac{d}{dt}\, c_{\text{IL-1βcomplex1}}(t) = k_{v3} \cdot c_{\text{IL-1βcomplex0}}(t) \cdot c_{\text{IL-1β}}(t) - k_{v4} \cdot c_{\text{IL-1βcomplex1}}(t) - k_{v5} \cdot c_{\text{IL-1βcomplex1}}(t) \cdot c_{\text{Tab/Tak1}}(t) \tag{32}$$

$$\frac{d}{dt}\, c_{\text{IL-1βcomplex2}}(t) = k_{v5} \cdot c_{\text{IL-1βcomplex1}}(t) \cdot c_{\text{Tab/Tak1}}(t) - k_{v6} \cdot c_{\text{IL-1βcomplex2}}(t) - k_{v7} \cdot c_{\text{IL-1βcomplex2}}(t) \tag{33}$$

$$\frac{d}{dt}\, c_{\text{IL-1βcomplex3}}(t) = k_{v7} \cdot c_{\text{IL-1βcomplex2}}(t) - k_{v8} \cdot c_{\text{IL-1βcomplex3}}(t) - k_{v9} \cdot c_{\text{IL-1βcomplex3}}(t) \tag{34}$$

$$\frac{d}{dt}\, c_{\text{JNK}}(t) = k_{v13} \cdot c_{\text{pJNK}}(t) - k_{v12} \cdot c_{\text{JNK}}(t) \cdot c_{\text{pMKK7}}(t) \tag{35}$$

$$\frac{d}{dt}\, c_{\text{MKK7}}(t) = k_{Sv27} \cdot c_{\text{pMKK7}}(t) \cdot c_{\text{phosphatase}}(t) - k_{v11} \cdot c_{\text{MKK7}}(t) \cdot c_{\text{Tak1*}}(t) \tag{36}$$

$$\frac{d}{dt}\, c_{\text{NFκB\_c}}(t) = k_{L4\_t2} \cdot c_{\text{IKKa\_IκBα\_NFκB}}(t) + k_{L24\_c6a} \cdot c_{\text{IκBα\_NFκB\_c}}(t) - k_{L5\_i1} \cdot c_{\text{NFκB\_c}}(t) - k_{L25\_a1} \cdot c_{\text{NFκB\_c}}(t) \cdot c_{\text{IκBα\_c}}(t) \tag{37}$$

$$\frac{d}{dt}\, c_{\text{NFκB\_n}}(t) = k_{L5\_i1} \cdot c_{\text{NFκB\_c}}(t) - k_{L11\_a1} \cdot V_{\text{Cyt:Nuc}} \cdot c_{\text{IκBα\_n}}(t) \cdot c_{\text{NFκB\_n}}(t) \tag{38}$$

$$\frac{d}{dt}\, c_{\text{pBim}}(t) = k_{Sv3} \cdot c_{\text{Bim}}(t) \cdot c_{\text{pJNK}}(t) - k_{Sv4} \cdot c_{\text{pBim}}(t) - k_{Sv5} \cdot c_{\text{pBim}}(t) \cdot c_{\text{Bcl-2}}(t) \tag{39}$$

$$\frac{d}{dt}\, c_{\text{phosphatase}}(t) = k_{Sv25} \cdot c_{\text{prophosphatase}}(t) \cdot c_{\text{pJNK}}(t) - k_{Sv26} \cdot c_{\text{phosphatase}}(t) \tag{40}$$

$$\frac{d}{dt}\, c_{\text{pJNK}}(t) = k_{v12} \cdot c_{\text{JNK}}(t) \cdot c_{\text{pMKK7}}(t) - k_{v13} \cdot c_{\text{pJNK}}(t) \tag{41}$$

$$\frac{d}{dt}\, c_{\text{pMKK7}}(t) = k_{v11} \cdot c_{\text{MKK7}}(t) \cdot c_{\text{Tak1*}}(t) - k_{Sv27} \cdot c_{\text{pMKK7}}(t) \cdot c_{\text{phosphatase}}(t) \tag{42}$$

$$\frac{d}{dt}\, c_{\text{prophosphatase}}(t) = k_{Sv26} \cdot c_{\text{phosphatase}}(t) - k_{Sv25} \cdot c_{\text{prophosphatase}}(t) \cdot c_{\text{pJNK}}(t) \tag{43}$$

$$c_{\text{Smac/DIABLO}}(t) = (1 + \tanh(30 * (c_{\text{Bax/Bak*}}(t) - 20))) * 50 \tag{44}$$

$$\frac{d}{dt}\, c_{\text{Smac-XIAP}}(t) = k_{v14} \cdot c_{\text{XIAP}}(t) \cdot c_{\text{Smac/DIABLO}}(t) \tag{45}$$

$$\frac{d}{dt}\, c_{\text{Tab/Tak1}}(t) = -k_{v5} \cdot c_{\text{Tab/Tak1}}(t) \cdot c_{\text{IL-1}\beta\text{complex1}}(t) \tag{46}$$

$$\frac{d}{dt}\, c_{\text{Tak1*}}(t) = k_{v9} \cdot c_{\text{IL-1}\beta\text{complex3}}(t) - k_{v10} \cdot c_{\text{Tak1*}}(t) \tag{47}$$

$$\begin{aligned}\frac{d}{dt}\, c_{\text{tBid}}(t) &= k_{v19} \cdot c_{\text{Bid}}(t) \cdot c_{\text{C8p18}}(t) - k_{Sv10} \cdot c_{\text{tBid}}(t) \\ &\quad - k_{Sv11} \cdot c_{\text{tBid}}(t) \cdot c_{\text{Bcl-2}}(t)\end{aligned} \tag{48}$$

$$\frac{d}{dt}\, c_{\text{X}}(t) = k_{v23} \cdot c_{\text{X\_mRNA}}(t) - k_{v24} \cdot c_{\text{X}}(t) \tag{49}$$

$$\frac{d}{dt}\, c_{\text{X\_mRNA}}(t) = k_{v21} \cdot c_{\text{NF}\kappa\text{B\_n}}(t) - k_{v22} \cdot c_{\text{X\_mRNA}}(t) \tag{50}$$

$$\frac{d}{dt}\, c_{\text{XIAP}}(t) = -k_{v14} \cdot c_{\text{XIAP}}(t) \cdot c_{\text{Smac/DIABLO}}(t) - k_{Sv18} \cdot c_{\text{XIAP}}(t) \cdot c_{\text{C3*}}(t) \tag{51}$$

$$V_{\text{Cyt:Nuc}} = \frac{V_{\text{Cytosol}}}{V_{\text{Nucleus}}} = 3 \tag{52}$$

Parameterization

The parameters of the NF-κB module (reactions labeled L_i) [4] as well as of the reactions of the TNFα/FasL model that were adopted without any modification (labeled Sv_i) [1] were all retained. Several parameters of the IL-1β signaling module could be adjusted according to information found in literature [14] and to the JNK immunblot (Fig. 4A) as explained in more detailed below. The remaining five of the 70 parameters (k_{v14}, k2_{v15}, k_{v16}, k_{v19}, k_{v20}) of the IL-1β/FasL model were manually curated in an iterative approach for reproduction of the available experimental data. All parameter values of the IL-1β/FasL model are listed in Table S2.

The IL-1β module is based on literature research where also some evidence for estimation of the parameters for formation of the IL-1β receptor complexes could be found. Jiang et al. investigated time-dependent interactions of important molecules involved in

IL-1β signaling and, thus, his data was used to estimate the values of the parameters k_{v1} - k_{v10} (Fig. S1) [14]. They found that Irak1 and Traf6 co-immunoprecipitate with the receptor IL-1R1 already one minute after IL-1β treatment, which corresponds to formation of IL-1β complex 1, and was sustained for about two hours. Co-immunoprecipitation of Irak1 and Traf6 with Tak1 and Tab correspond to formation of IL-1β complex 2. This interaction was observed two minutes after IL-1β stimulation and disappeared about two hours after treatment. Cytosolic Tab could be observed five minutes after IL-1β treatment corresponding to formation of IL-1β complex 3 and active Tak1 (Tak1*) could be detected two minutes after stimulation and was maintained over at least 60 min.

Parameters of pMKK7-induced JNK phosphorylation (k_{v12}) and dephosphorylation of pJNK (k_{v13}) (Fig. S2) were adapted to the JNK immunblot (Fig. 4A). JNK is phosphorylated within the first 30 min after IL-1β stimulation with the peak already at 10 min after stimulation. After 1 h the induction was clearly reduced, but remained at a basal level for at least 12 h.

References

[1] Schlatter R, Schmich K, Lutz A, Trefzger J, Sawodny O, et al. (2011) Modeling the TNFα-induced apoptosis pathway in hepatocytes. PloS One 6: e18646. doi:10.1371/journal.pone.0018646.

[2] Mosmann T (1983) Rapid colorimetric assay for cellular growth and survival: application to proliferation and cytotoxicity assays. J Immunol Methods 65: 55–63.

[3] Schreiber E, Matthias P, Müller MM, Schaffner W (1989) Rapid detection of octamer binding proteins with 'mini-extracts', prepared from a small number of cells. Nucleic Acids Res 17: 6419.

[4] Lipniacki T, Paszek P, Brasier AR, Luxon B, Kimmel M (2004) Mathematical model of NF-kappaB regulatory module. J Theor Biol 228: 195–215. doi:10.1016/j.jtbi.2004.01.001.

[5] Muñoz Pinedo C, Guío-Carrión A, Goldstein JC, Fitzgerald P, Newmeyer DD, et al. (2006) Different mitochondrial intermembrane space proteins are released during apoptosis in a manner that is coordinately initiated but can vary in duration. Proc Natl Acad Sci U S A 103: 11573–11578. doi: 10.1073/pnas.0603007103.

[6] Rehm M, Düssmann H, Prehn JHM (2003) Real-time single cell analysis of Smac/DIABLO release during apoptosis. J Cell Biol 162: 1031–1043. doi: 10.1083/jcb.200303123.

[7] Strasser A, Jost PJ, Nagata S (2009) The many roles of FAS receptor signaling in the immune system. Immunity 30: 180–192. doi: 10.1016/j.immuni.2009.01.001.

[8] Du C, Fang M, Li Y, Li L, Wang X (2000) Smac, a mitochondrial protein that promotes cytochrome c-dependent caspase activation by eliminating IAP inhibition. Cell 102: 33–42.

[9] Gao Z, Tian Y, Wang J, Yin Q, Wu H, et al. (2007) A dimeric Smac/diablo peptide directly relieves caspase-3 inhibition by XIAP. Dynamic and cooperative regulation of XIAP by Smac/Diablo. J Biol Chem 282: 30718–27. doi: 10.1074/jbc.M705258200.

[10] Jost PJ, Grabow S, Gray D, McKenzie MD, Nachbur U, et al. (2009) XIAP discriminates between type I and type II FAS-induced apoptosis. Nature 460: 1035–1039. doi: 10.1038/nature08229.

[11] Daniel S, Arvelo MB, Patel VI, Longo CR, Shrikhande G, et al. (2004) A20 protects endothelial cells from TNF-, Fas-, and NK-mediated cell death by inhibiting caspase–8 activation. Blood 104: 2376–2384. doi: 10.1182/blood-2003-02-0635.

[12] Kallenberger SM, Beaudouin J, Claus J, Fischer C, Sorger PK, et al. (2014) Intra- and interdimeric caspase-8 self-cleavage controls strength and timing of CD95-induced apoptosis. Sci Signal 7: ra23. doi: 10.1126/scisignal.2004738.

[13] Hoops S, Sahle S, Gauges R, Lee C, Pahle J, et al. (2006) COPASI—a COmplex PAthway SImulator. Bioinformatics (Oxford, England) 22: 3067–3074. doi: 10.1093/bioinformatics/btl485.

[14] Jiang Z, Ninomiya-Tsuji J, Qian Y, Matsumoto K, Li X (2002) Interleukin-1 (IL-1) receptor-associated kinase-dependent IL-1-induced signaling complexes phosphorylate TAK1 and TAB2 at the plasma membrane and activate TAK1 in the cytosol. Mol Cell Biol 22: 7158–7167. doi: 10.1128/MCB.22.20.7158.

[15] Keyse SM (2000) Protein phosphatases and the regulation of mitogen-activated protein kinase signalling. Curr Opin Cell Biol 12: 186–192.

[16] Hamdi M, Kool J, Cornelissen-Steijger P, Carlotti F, Popeijus HE, et al. (2005)

DNA damage in transcribed genes induces apoptosis via the JNK pathway and the JNK-phosphatase MKP-1. Oncogene 24: 7135–7144. doi: 10.1038/sj.onc.1208875.

[17] Ninomiya-Tsuji J, Kishimoto K, Hiyama A, Inoue J, Cao Z, et al. (1999) The kinase TAK1 can activate the NIK-I kappaB as well as the MAP kinase cascade in the IL-1 signalling pathway. Nature 398: 252–256. doi: 10.1038/18465.

[18] Letai AG (2008) Diagnosing and exploiting cancer's addiction to blocks in apoptosis. Nat Rev Cancer 8: 121–132. oi: 10.1038/nrc2297.

[19] Goldstein JC, Waterhouse NJ, Juin P, Evan GI, Green DR (2000) The coordinate release of cytochrome c during apoptosis is rapid, complete and kinetically invariant. Nat Cell Biol 2: 156–162. doi: 10.1038/35004029.

[20] Schmich K, Schlatter R, Corazza N, Sá Ferreira K, Ederer M, et al. (2011) Tumor necrosis factor α sensitizes primary murine hepatocytes to Fas/CD95-induced apoptosis in a Bim- and Bid-dependent manner. Hepatology 53: 282–292. doi: 10.1002/hep.23987.

[21] Düssmann H, Rehm M, Concannon CG, Anguissola S, Würstle M, et al. (2010) Single-cell quantification of Bax activation and mathematical modelling suggest pore formation on minimal mitochondrial Bax accumulation. Cell Death Diff 17: 278–290. doi 10.1038/cdd.2009.123.
